# The Food Environment Toolbox: Developing and Piloting a Suite of Tools to Measure Food Environments in Low- and Middle-Income Countries

**DOI:** 10.1016/j.cdnut.2025.107444

**Published:** 2025-04-16

**Authors:** Shauna M Downs, Wiktoria Staromiejska, Neha Bakshi, Serey Sok, Nyda Chhinh, Punleu Thou, Nayelin Phorn, To Chen, Selena Ahmed, Elizabeth L Fox, Anna Herforth, Suparna Ghosh-Jerath

**Affiliations:** 1 Department of Health Behavior, Society and Policy, Rutgers School of Public Health, Newark, NJ, United States; 2 Department of Nutrition, The George Institute for Global Health, Delhi, India; 3 Research Office, Royal University of Phnom Penh, Russian Federation Boulevard, Khan Toul Kork, Cambodia; 4 Department of Economic Development, Faculty of Development Studies, Royal University of Phnom Penh, Russian Federation Boulevard, Khan Toul Kork, Cambodia; 5 Independent Consultant, Phnom Penh, Cambodia; 6 Child Rights Foundation, Sangkat Dang Kor, Khan Dang Kor, Phnom Penh, Cambodia; 7 Periodic Table of Food Initiative, American Heart Association, USA; 8 Department of Public & Ecosystem Health, Cornell University College of Veterinary Medicine, Ithaca, NY, United States; 9 Division of Human Nutrition and Health, Wageningen University and Research, Wageningen, The Netherlands

**Keywords:** food environments, low- and middle-income countries, tools, external food environment, personal food environment, lived experiences, community mapping

## Abstract

**Background:**

Measuring food environments in low- and middle-income countries (LMICs) can inform and support the design of interventions aimed at improving diets and reducing malnutrition in all its forms. Most food environment measurement tools have been developed for high-income countries, however, and do not sufficiently capture the diverse and dynamic food environments in LMICs.

**Objectives:**

The objective of this study was to develop, modify, implement, and refine a suite of tools to measure the different dimensions of diverse food environments in LMICs.

**Methods:**

Through an iterative process, we: *1*) identified the food environment dimensions to be measured; *2*) identified existing tools using literature searches; *3*) modified and developed tools based on feedback from team members of study settings, an expert advisory board, and workshops with key food environment experts in India and Cambodia; *4*) implemented the tools by piloting the suite in rural, peri-urban, and urban settings in India and Cambodia; and *5*) finalized tools based on feedback from experts and our tool piloting implementation and analysis experience.

**Results:**

Overall, we included 7 tools in the finalized Toolbox (Participatory Mapping, Seasonal Food Availability Calendar, Food Environment Perceptions Survey, Community Food Environment Mapping, Market Mapping, In-depth Vendor Assessment, and a Cost of a Healthy Diet data collection protocol), all of which were rated positively by workshop participants. On the basis of piloting experiences, the tools were relatively easy to implement in the field. Apart from the Seasonal Food Availability Calendar being better suited to rural or peri-urban settings and the In-depth Vendor Assessment being less suitable for large formal supermarkets, we found that the tools were feasible and useful across pilot settings in India and Cambodia.

**Conclusions:**

The suite of tools included in the Food Environment Toolbox can be used to measure diverse food environments in LMICs, with minimal anticipated adaptations across contexts.

## Introduction

Food environments—defined as the “consumer interface within the food system that encompasses the availability, affordability, convenience, quality and promotion, and sustainability of foods and beverages in wild, cultivated, and built spaces” [1]—influence our choices regarding what to eat and where to source it [2]. In low- and middle-income countries (LMICs), food environments are multifaceted [1,3] and are quickly changing over time [4]. Over the past 50 y, LMICs have seen transformations in the way people access food, moving from smallholder and subsistence farmers growing food for household consumption and local market purchases to buying food from greater distances, with supermarkets rapidly proliferating, especially in Asian and Latin American markets [[5], [6], [7]]. This shift has been accompanied by significant economic growth, yet these economic gains have not resulted in comparable improvements in nutrition and health outcomes [8]. Most countries globally—and LMICs in particular—experience burdens of >1 form of malnutrition, including undernutrition (stunting, underweight, and wasting), overweight, obesity, diet-related noncommunicable disease, and micronutrient deficiencies [8,9]. These multiple burdens of malnutrition have often been linked with changes in food environments and their impact on dietary intakes at the population level [4]. In some settings, this has been specifically associated with a reduced prevalence of undernutrition (but not micronutrient deficiencies) among children [[10], [11], [12], [13]].

Given the role that food environments play in shaping dietary behavior, their measurement in LMICs can assist with monitoring and surveillance of the current context and better inform context-based interventions aimed at improving diets and reducing the burdens of malnutrition. Many of the existing tools designed to assess food environments were originally developed for, and applied to, high-income countries, limiting their applicability in the diverse and dynamic contexts of LMICs [14]. In LMICs, people access food from both natural (i.e., wild and cultivated) and built food sources [1] as well as through the exchange of food within their social networks (i.e., kin and community) [3]. For that reason, there is a need for tools that can both objectively and qualitatively assess the different food environment dimensions (e.g., food availability, affordability, quality, etc.) across the different food environment types where people access food. Tools need to capture both the external or objective food environments, as well as consumers’ lived experiences interacting with their food environments to determine how these different food environment dimensions influence which foods people acquire, purchase, or consume.

In recognition of the need to develop tools that are better suited to measuring food environments in LMICs, several teams globally are working on developing food environment tools [[15], [16], [17], [18], [19], [20], [21]]. The Food Environment Toolbox contributes to this work by providing a “one-stop shop” for researchers, planners, and practitioners to identify field and user-friendly, nonresource-intensive, open-access, and scalable tools to measure the various dimensions of the food environment in diverse LMIC contexts. The overarching aims of the Food Environment Toolbox are to: *1*) provide guidance on the selection and application of food environment tools in LMICs that describe different food environment dimensions; *2*) advance a standardized, systematic, and scientific approach of measuring and assessing food environments in LMICs; *3*) enable the examination of food environments over time using standardized tools at multiple time points; and *4*) examine the relationship between food environments and food security, diet quality, nutrition, and health outcomes. This article aims to describe the development, modification, implementation, and refinement of tools for the Food Environment Toolbox. By documenting this process, we provide food environment researchers, planners, and practitioners with an overview of tools and methodologies that they can choose from, as well as implementation strategies that might be most relevant to address their unique research questions around food environment assessment.

## Methods

**FIGURE 1:**
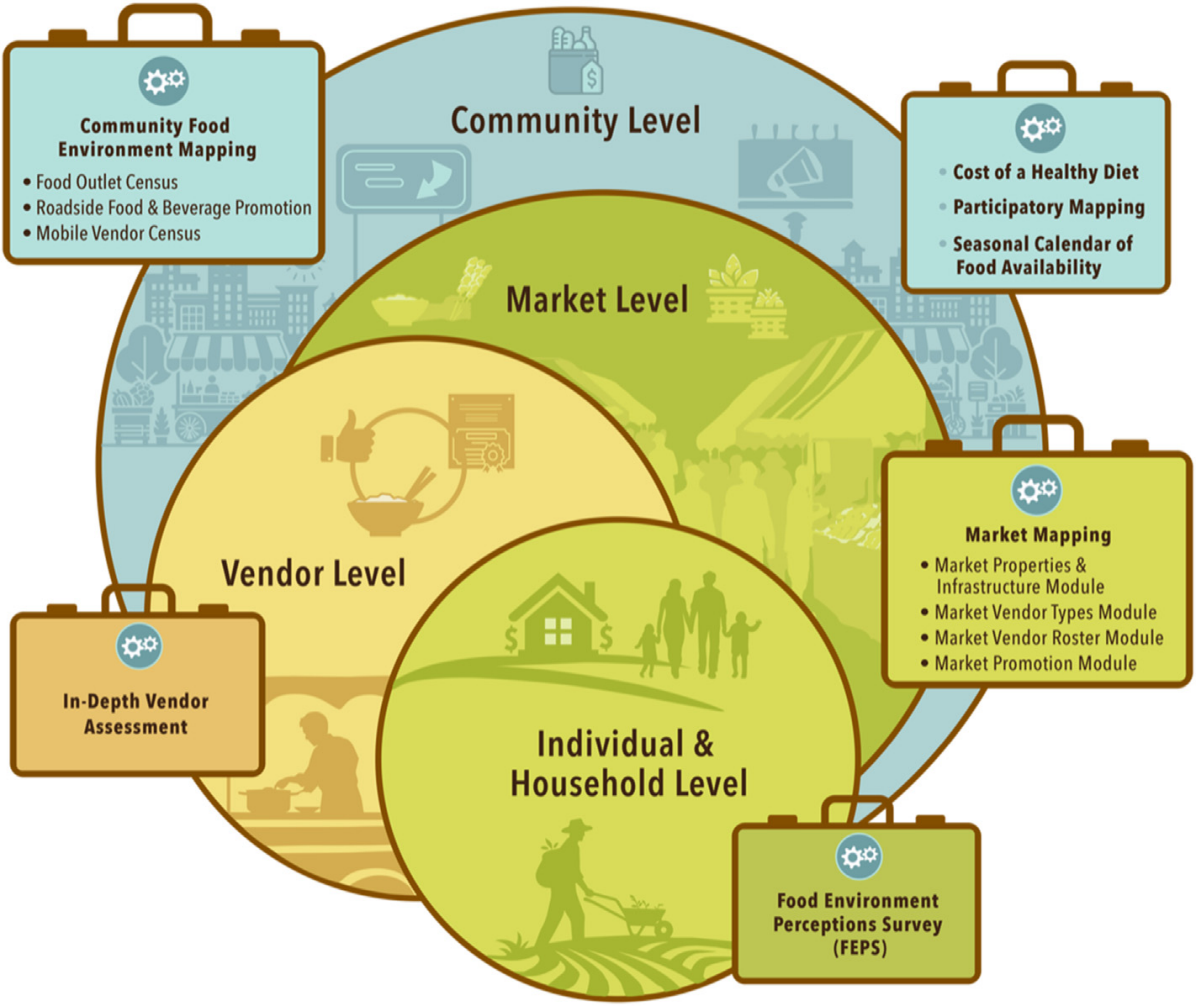
An overview of the tools included in the Food Environment Toolbox and their level of data collection.

This article describes: *1*) the development of new and modification of existing food environment tools for LMICs, and *2*) the pilot testing of the tools in India and Cambodia as case studies. The 7 tools that were included in the final Food Environment Toolbox include: Participatory Mapping, Seasonal Food Availability Calendar, Food Environment Perceptions Survey (FEPS), Community Food Environment Mapping (Food Outlet Census module, Roadside Food and Beverage Promotions module and Mobile Vendor Census module), Market Mapping, In-depth Vendor Assessment, and a Cost of a Healthy Diet (CoHD) data collection protocol. The implementation of each of these tools is described in more detail below. Together the tools capture data at the community (i.e., village or neighborhood), market (i.e., key markets that consumers acquire food from), vendor (i.e., the individual vendors that consumers acquire food from), and individual and household level (i.e., factors that influence how consumers interact with their food environments) (Figure 1). The final tools can be found on the Food Environment Toolbox website (https://sites.rutgers.edu/food-environment-Toolbox/).

We obtained Institutional Review Board approval from Rutgers University, The George Institute for Global Health, India Institutional Ethics Committee, and the National Ethics Committee for Health Research in Cambodia. Finally, we obtained approvals from local authorities in both countries to complete the fieldwork. The research team from Rutgers University worked with local scholars from The George Institute for Global Health and Royal University of Phnom Penh for the data collection and fieldwork in India and Cambodia, respectively.

### Study design

**FIGURE 2:**
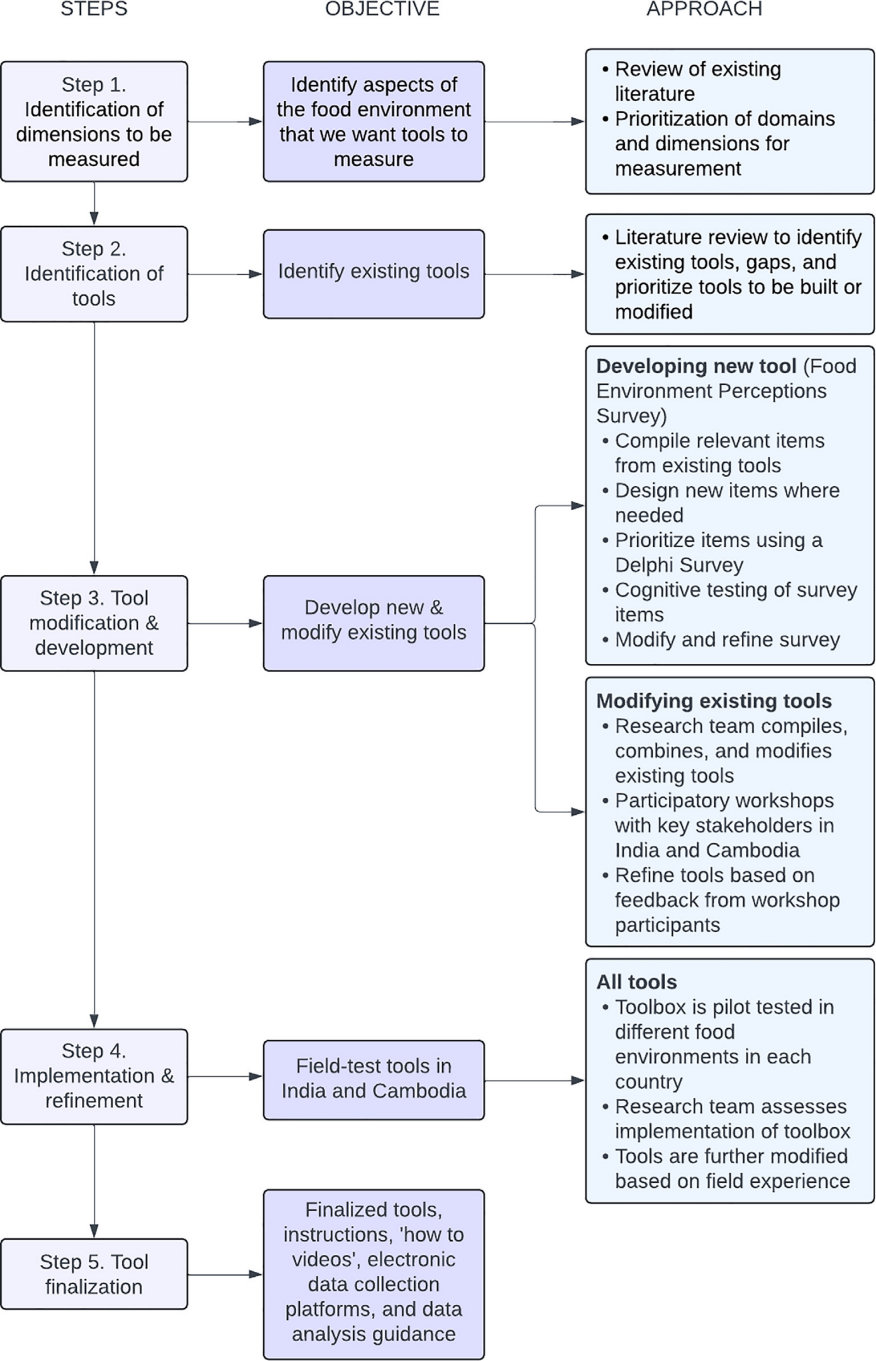
Overview of approach to the Food Environment Toolbox development.

Figure 2 provides an overview of the process of refining the tools included in the Food Environment Toolbox. We first identified the dimensions of the food environment to be measured by the tools. We then identified existing tools that could be used or adapted, and new tools that needed to be developed through reviewing existing literature and engagement with our study team. We then modified the existing tools based on feedback from our study team, the Food Environment Toolbox Advisory Board (i.e., a group of 5 global food environment experts), and food environment experts participating in consultative workshops in India and Cambodia. We also developed a new tool (i.e., FEPS) to assess people’s food environment perceptions and lived experience interacting with their food environments. The development and application of the FEPS are reported in more detail elsewhere [22]. We piloted all the tools in the Toolbox and finalized them based on our field experiences. Except for 1 tool [i.e., Produce Desirability (ProDes) tool], all tools that were initially selected, reviewed by experts, and piloted, were included—in modified forms—in the final Toolbox.

### Step 1: identification of dimensions of the food environment to be measured

Food environment dimensions included food availability (type and diversity of foods on offer), food affordability (food prices, alone and in comparison, to income and expenditures), food properties (safety, quality, appeal, convenience, and sustainability), vendor properties (location and type), and food messaging (promotion, advertising, and information about food) [1,5]. These dimensions interact with individual factors to influence diet, including economic (income and purchasing power), cognitive (information and knowledge), aspirational (desires, values, preferences), and situational (home and work environment, mobility, location, time) factors [5,23]. Although individual factors are not themselves part of the food environments, they have a powerful impact on the perceived food environment. For example, 1 person may perceive fruits as unaffordable, whereas another may perceive them as affordable, owing to different combinations of individual factors such as income, tastes knowledge, and aspirations (i.e., the value an individual places on fruits and the ability to pay for them, in comparison to their objective market cost). We sought tools for measuring both the objective and perceived food environments. Elsewhere in the literature, the objective food environment has been referred to as “external” and aspects of the perceived food environment have been referred to as the “personal” food environment [14]. We also sought tools for measuring the different types of food environments people access food from in LMICs, including natural (i.e., wild and cultivated), built (i.e., formal and informal), wild, supplemental food assistance, and kin and community [1,3].

### Step 2: identification of tools

#### Review of literature

We reviewed the literature to examine existing tools that could be used to measure and assess the different dimensions of food environments across diverse contexts as part of our previous work [1,24]. We subsequently built on those reviews and conducted additional targeted searches of the literature to identify additional tools that had been developed to measure food environments. We engaged in informal communications with food environment researchers whom we knew had developed or were in the process of developing tools, to add the tools to our repository of existing tools. In addition to members of our research team reviewing the tools, we obtained feedback from members of the Food Environment Toolbox Advisory Board, comprising experts in food environment conceptualization and measurement in LMICs. The existing tools included: United States Agency for International Development (USAID) Advancing Nutrition Market-based Food Environment Assessments [25], Software Tools for Calculating the CoHD from Food Prices for Nutrition [26], Global Alliance for Improved Nutrition (GAIN) and USAID EatSafe vendor observation checklist [27], the Environmental Profile of a Community’s Health tool [28], Multisectoral Food and Nutrition Security project Cambodia [29], the International Network for Food and Obesity/noncommunicable diseases Research, Monitoring and Action Support modules [30], and the World Food Programme’s Market Functionality Index [31].

### Step 3: tool development

#### Refinement of tools based on our team’s experience

Once an initial draft of each tool was developed, members of our research team modified existing tools (both those developed by members of our team and other experts in the field), as relevant, building on our methodological experience characterizing food environments in various contexts, field-testing tools, and discussing their benefits and drawbacks in recent food environment measurement workshops. In particular, we began by building on several of the USAID Advancing Nutrition’s Market-based Food Environment Assessments Guidelines [25] and the lessons learned from the pilot testing of those tools in different countries [32] based on our study team’s participation in that work in technical and advisory roles. Members of our team used early versions of some of the Food Environment Toolbox tools as part of another food environment project, which was previously conducted in Cambodia, which provided additional information and understanding regarding gaps in the tools and challenges in terms of their implementation in the field [33,34]. Supplemental Table 1 provides an overview of how the different existing tools informed the final tools included in the Toolbox.

The process of modifying existing tools prior to conducting consultative workshops and piloting took place over 1.5 y and was iterative. The process included taking questions from existing tools, refining them, and combining them with questions from other tools to create versions that captured relevant information while simultaneously being user-friendly in the field.

There were 2 key classification systems that were used across the tools and that we refined throughout the piloting stage: *1*) food group classifications; and *2*) food outlet/vendor type classifications. As part of this process, we examined existing food group classifications (e.g., Healthy Diet Basket [35], Diet Quality Questionnaire [36], Global-Diet Quality Score [37], Minimum Dietary Diversity for Women [38], and the NOVA classifications related to nature and extent of food processing [39]) to inform the food group classifications that would best be used across the Toolbox as well as being suitable for LMIC contexts. Most of these classifications were designed to measure diets—a task requiring a larger number of food groups than may be necessary for assessing foods in the food environment. The Healthy Diet Basket [35] was designed to assess foods in the food environment, but only to assess healthy diets, so its 6 food groups do not cover discretionary or unhealthy foods in sufficient detail to assess the food environment. We therefore used the food groupings in these published classification systems at a level of aggregation that is fit for the purpose of understanding the types of healthy and less healthy foods in food environments. The food outlet/vendor types were initially informed by our teams’ experience working in diverse food environments in LMICs and were subsequently modified based on feedback during workshops and our experiences during pilot testing.

#### Workshops with key food environment, public health, and food system experts

After the initial modifications of tools by members of the research team, we conducted participatory consultative workshops with food environment, public health, and food systems researchers, planners, practitioners from government and nongovernmental organizations (NGOs), and members of community-based organizations in India (November 2023) and Cambodia (March 2024). Throughout both workshops, detailed notes were taken to capture the discussions related to the tools and the suggestions for changes. A total of 20 participants attended the 2-d workshop in India from universities (*n* = 7), research institutions (*n* = 9), UN agencies (*n* = 2), and NGOs (*n* = 2). The workshop content included: providing a background on food environments and the benefits to measuring them; an overview of each of the proposed tools; and a discussion of the merits of each of the tools, changes that should be made to strengthen them, and their gaps (see Appendix A for workshop agenda). Thirteen workshop participants in India responded to postworkshop surveys to provide additional feedback on the tools (see Appendix B for workshop survey) and to rate the tools based on their overall assessment of each tool, the food groups used, as well as specific questions related to the content of each tool. The workshop participants were asked to rate the tools on a 3-point scale from negative to positive. Response options differed based on the specific questions. Negative response options included: "inadequately covered," "no," "disagree," "unsatisfactory," "difficult," "lengthy," and "speculative." Positive response options included: "adequately covered," "yes," "agree," "well presented," "easy," "short," and "definite." Immediately after the workshop, we developed a detailed list of all changes that needed to be made to the tools prior to their piloting in India.

In Cambodia, we conducted a 1-d workshop with 28 participants representing universities (*n* = 9), research institutions (*n* = 2), UN agencies (*n* = 2), NGOs (*n* = 12), and government (*n* = 3). Given differences in the cultural context, we used a different approach for the workshop (see Appendix C for workshop agenda). We began the workshop by providing a background on food environments, the benefits of measuring them, as well as a short overview of each of the tools to all the workshop participants together. Workshop participants then worked in small groups to provide detailed feedback on each tool. Each small group provided feedback on hard copies of each of the tools to reflect changes that they perceived would strengthen the tools given each tool’s purpose. A member of our research team facilitated each of the small group discussions, and a representative from each of the groups presented their feedback to the larger group. Thirteen workshop participants completed the postworkshop survey to assess the different tools. The workshop survey was similar to the one used in India, but was revised to reduce its length, target feedback on outstanding queries we had about the tools, and enhance respondent granularity through scale expansion (see Appendix D for a copy of the workshop survey). Workshop participants were asked to respond to each question on a 5-point scale ranging from "strongly disagree" to "strongly agree." Response options of "strongly agree" or "agree" were described as positive responses, and "disagree" and "strongly disagree" were described as negative responses in the results. After each of the workshops was completed, we made additional changes to the tools to strengthen them prior to piloting them.

### Step 4: implementation and feedback

In both India and Cambodia, we piloted the tools in rural, peri-urban, and urban (low- and high-income) settings. More specifically, in India, we piloted the Toolbox in urban areas of Delhi and Bengaluru, and peri-urban (Narayanganj block of Mandla district, Madhya Pradesh) and villages of rural (Bicchia block of Mandla district in Madhya Pradesh). However, the Participatory Mapping and Seasonal Food Availability Calendar were only conducted in the rural and urban settings. In Cambodia, we piloted the tools in urban areas of Phnom Penh (Khan Russey Keo and Khan Boeng Keng Kang), a peri-urban area outside of Phnom Penh (Khan Kamboul district), and a rural setting in Kampong Cham province (Kampong Siem district).

In India, the initial pilot testing in Mandla district was conducted in February–March 2024. We made additional changes to the tools based on our piloting experience prior to completing the pilot testing in India between April and May 2024. In India, all data collectors (*n* = 7) had backgrounds in nutritional sciences. In Cambodia, all piloting of the tools took place between April and May 2024. In Cambodia, data collectors (*n* = 9) did not have direct backgrounds in nutrition, but most had participated in previous studies examining food environments with members of our study team.

Our approach to data collection during the piloting was tool dependent (see Table 1 [22] for an overview). Focus group discussions (FGDs) were conducted as part of the Participatory Mapping and Seasonal Calendar of Food Availability tools, tablets using the KoboToolbox platform were used to collect data for Community and Market Mapping tools, as well as the In-depth Vendor Assessment, and paper-based forms were used to collect food price and quality/desirability data for the CoHD and ProDes tools, respectively. Given that the goal of the research was to gain insight into the feasibility of implementing the different tools, the sampling approaches differed from one study site to another. In an iterative process, and after each day of piloting in the field, the team debriefed about what worked and did not work, and brainstormed changes that could be made to make the tools, or their programming in the tablet, more streamlined. We then piloted the proposed changes during the next day of data collection.

**TABLE 1:** An overview of the aims and approach to pilot data collection for each of the Food Environment Toolbox tools.

Tool name	Aims	Approach to data collection	Sampling approach	Pilot setting	Sample size	Mode of data collection	Mode of data analysis
Participatory Mapping	• Identify the types of food environments people are accessing in their community	• Focus group discussion	• Convenience	India^1^	*n* = 34, 4 FGDs	• Drawing of map • Recording of FGD	• Qualitative
				Cambodia	*n* = 25, 4 FGDs		
Seasonal Food Availability Calendar	• Assess seasonal availability of foods in the community • Assess perceived changes to food availability	• Focus group discussion	• Purposive	India^1^	*n* = 41, 6 FGDs	• Recording of FGD • Monthly recording of food availability on paper	• Qualitative
				Cambodia	*n* = 52, 8 FGDs		
Food Environment Perceptions Survey^2^	• Assess people’s perceptions of their food environment	• Enumerator-administered survey	• Purposive	India	*n* = 43	• Tablet	• Quantitative
				Cambodia	*n* = 60		
Community Food Environment Mapping	• Types of food outlets people are accessing • Types of foods sold and promoted in their community	• Observational	• Census	India	*n* = 20	• Tablet	• Quantitative • Spatial
				Cambodia	*n* = 326		
Market Mapping	• Types of vendors people have access to in markets • Types of foods sold and promoted in markets	• Observational	• Census • Subsampling	India	*n* = 193 vendors; *n* = 6 markets	• Tablet	• Quantitative
				Cambodia	*n* = 137 vendors; *n* = 4 markets		
In-depth Vendor Assessment	• Assess various properties of foods being sold and the vendors that sell them	• Observational	• Purposive • Subsampling	India	*n* = 42	• Tablet	• Quantitative
				Cambodia	*n* = 74		
Cost of a Healthy Diet	• Assess the cost of a healthy diet meeting dietary guidelines	• Observational	• Convenience	India	*n* = 2 communities^3^ *n* = 4 communities^4^	• Paper-based form	• Quantitative
				Cambodia			
Produce Desirability tool	• Assess sensory properties of fresh foods	• Observational	• Purposive	India^5^	*n* = 2 communities *n* = 4 total foods	• Paper-based form	• Quantitative
				Cambodia	*n* = 4 communities *n* = 147 total foods		

Abbreviation: FGD, focus group discussion.

1 Only conducted in rural and urban settings in India.

2 The piloting experience of the Food Environment Perceptions Survey (FEPS) is reported elsewhere [22].

3 Food prices were collected for 16–29 unique food items per community.

4 Food prices were collected for 71–98 unique food items per community.

5 Data collection ceased before piloting was complete. We attempted to use the tool in focus group discussions; however, a lack of consensus remained among participants in their ratings of foods.

### Step 5: tool finalization

After piloting in both countries, we made any remaining changes that we deemed necessary based on our field experience. We then created “how-to” videos explaining each step of implementing the tools as well as written data analysis guidance.

### Data analysis

We analyzed survey data from workshop participants using descriptive statistics in Excel (version 16.88, 2024). We also analyzed notes from the workshops, team meetings, and piloting debriefs to identify key changes that were made to the tools throughout the tool development, modification, refinement, and implementation process. FGD recordings were reviewed and thematically summarized to describe any anticipated challenges in implementing the tools. For example, we elicited information about how participants would assess the quality of fruits, vegetables, and animal-source foods in the Participatory Mapping FGD to inform the criteria used to assess quality in the ProDes tool. Information from the FGDs informed modifications to the tool and its eventual exclusion from the Toolbox. In addition, we analyzed the data analysis protocols of the piloted tools to provide additional insights into the applicability across rural, peri-urban, and urban settings as well as the data analysis guidance we proposed for the tools. We provide information about the process as well as the changes made to each of the tools, below, in the Results section.

## Results

### Food Environment Toolbox overall

**FIGURE 3:**
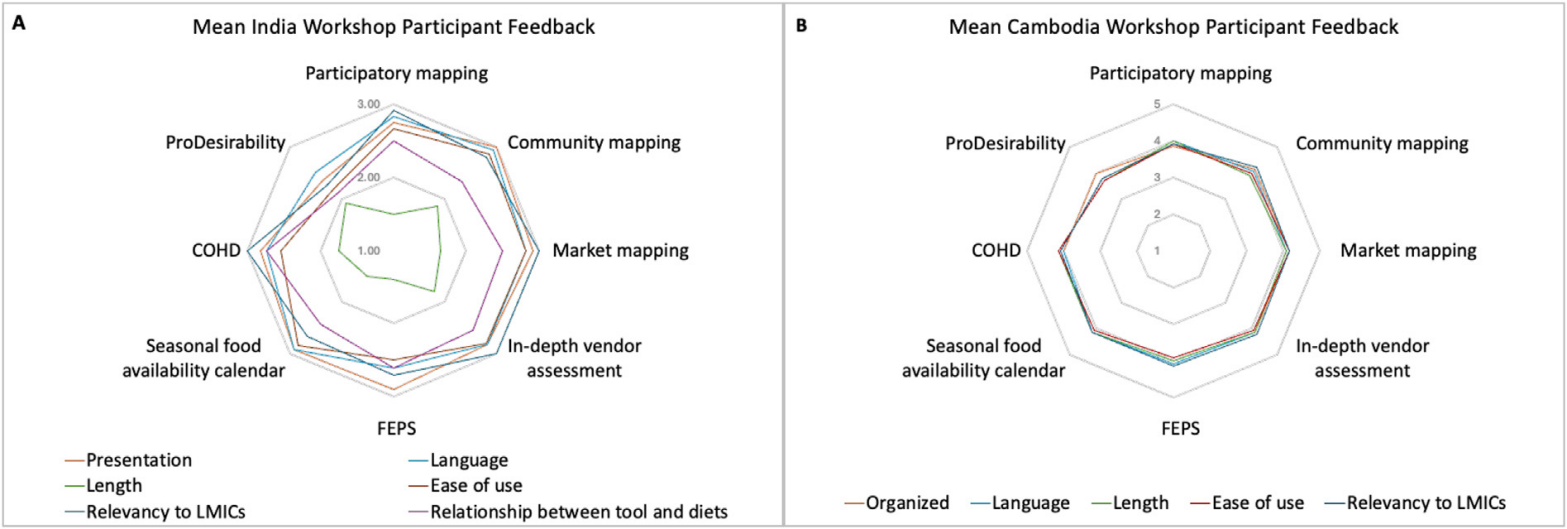
India^1,2^ and Cambodia^3^ workshop participants’ overall feedback on each of the tools. ^1^The scores from the workshop in India were rated from 1 (viewed negatively) to 3 (viewed positively). ^2^The scores for tool length in India ranged from 1 (very lengthy/somewhat lengthy) to 3 (short). The lengthy and somewhat lengthy response options were collapsed to enable their display on a 3-point scale. ^3^The scores from the workshop in Cambodia were rated from 1 (viewed negatively) to 5 (viewed positively). CoHD, cost of a healthy diet; FEPS, Food Environment Perceptions Survey; LMIC, low- and middle-income country.

Overall, workshop participants rated the tools relatively positively (Figure 3); however, the length of the tools was rated more negatively by workshop participants in India (panel A) as compared with Cambodia (panel B), where revised versions of the tools were presented in the workshop. In India, the Community Mapping and In-depth Vendor Assessments were rated the most positively; in Cambodia, the In-depth Vendor Assessment was rated most positively.

### Selection of food group classifications for Toolbox

Throughout each stage of tool development and implementation, the food group classifications were refined based on feedback from experts, workshop participants, and field experience. Most of the participants indicated that the food groups were appropriate in the postworkshop survey (94% in India and 92% in Cambodia). Moreover, in Cambodia, 92% of participants agreed that discretionary foods (i.e., optional foods that are not an essential part of the diet in terms of meeting nutrient needs) were relevant to include in the price data collection in an LMIC context. The final food group classifications can be found in Supplemental Table 2.

### Selection of food outlet/vendor classifications for Toolbox

In the initial versions of the tools, each tool used different food outlets and vendor types. However, after receiving feedback from workshop participants in India, we made them consistent across the tools. By the time we conducted the workshop in Cambodia, we had made significant changes to the food outlet/vendor types, and 92% of participants agreed/strongly agreed that they were sufficiently comprehensive. In the workshop in Cambodia, we also received feedback to add a higher-level classification of food outlets and vendor types which could facilitate data analysis. We subsequently added these (i.e., food service outlets/vendors, grocery retailer, specialty shop or vendor, wholesaler, supplemental food assistance) to the tools prior to piloting the tools in urban India and in all settings in Cambodia. For the FEPS tool, we also added a question to indicate whether the purchase was made online, by phone, or in person based on how common these transactions were in our pilot settings. Only minor adjustments to the vendor types were made after the pilot testing in Cambodia. Supplemental Table 3 provides an overview of the final food outlet/vendor classifications.

### Participatory Mapping

The Participatory Mapping tool was designed to describe the different food environments that community members access food from and which foods they access from those spaces. Overall, workshop participants in both countries viewed the Participatory Mapping tool positively, apart from their assessments about the length of the tool. When we piloted the tool during the FGD, it took between 60 and 90 min to complete in India and 60 and 85 min to complete in Cambodia. To reduce the amount of time to complete the FGD, we removed 2 sections (the questions that helped to inform the implementation of ProDes and questions related to food promotions in the community), which allowed for a more streamlined version of the tool. We also removed several questions in each of the sections to ensure the discussions flowed as well as possible.

**FIGURE 4:**
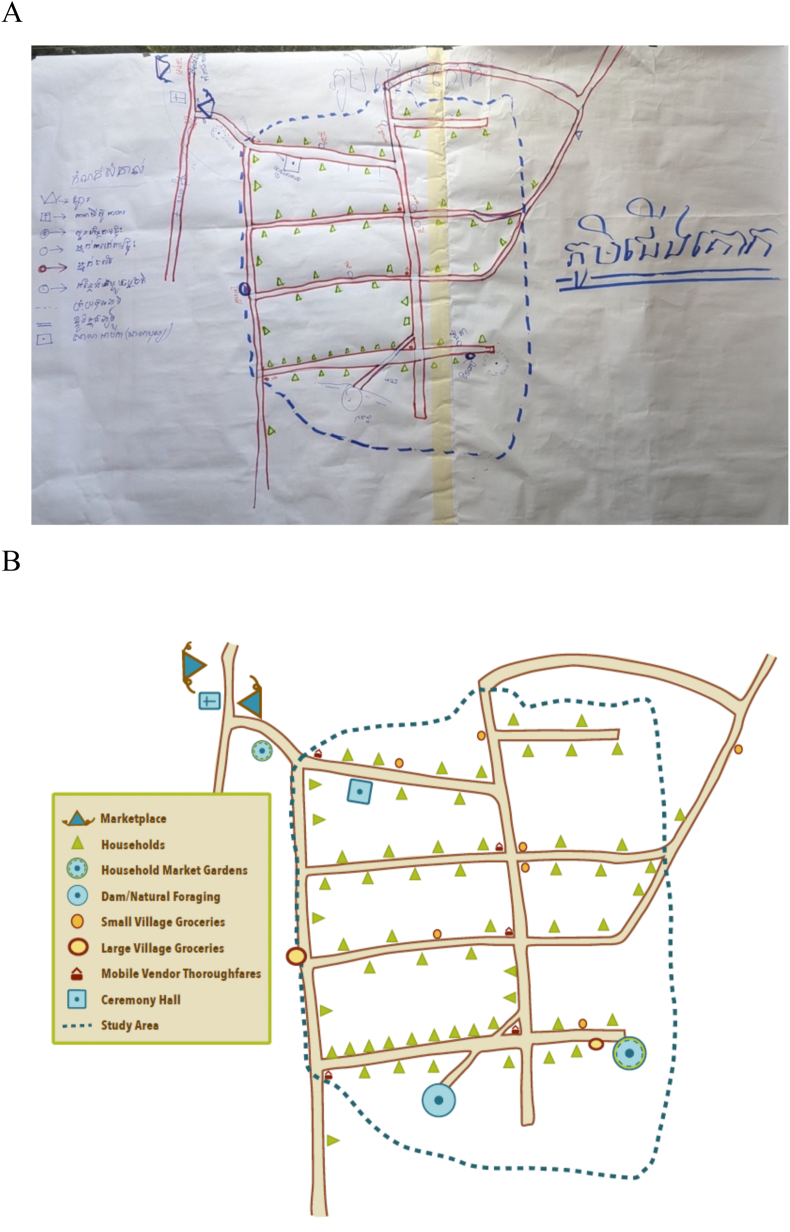
An example of a map drawn by Participatory Mapping participants during piloting in Cambodia. (A) Original map. (B) Digitized map.

During the piloting of the tool, facilitators observed that FGD participants were engaged in the process and were able to depict the maps of their food environments well (see Figure 4 for an example). In India, we started with an existing map of the community and FGD participants superimposed places where they accessed food. However, it was confusing for participants to locate specific food sites on the aerial map. Beginning with a blank sheet of paper was more feasible. In Cambodia, a sheet of paper that had the main roads outlined a priori was used as a starting point for the mapping. This allowed participants to engage with the relative location of different food sites rather than spending time on accurately placing the food sites.

After analyzing data collected in the pilots, we found that participants accessed several different food environment types across all settings in India and Cambodia. Table 2 depicts the food environment types accessed in the rural and urban settings in India and the foods available in each of those settings. Rural FGD participants in both India and Cambodia accessed a wide array of food environments: built, supplemental, kin and community, cultivated and wild; urban FGD participants accessed more built food environment types.

**TABLE 2:**
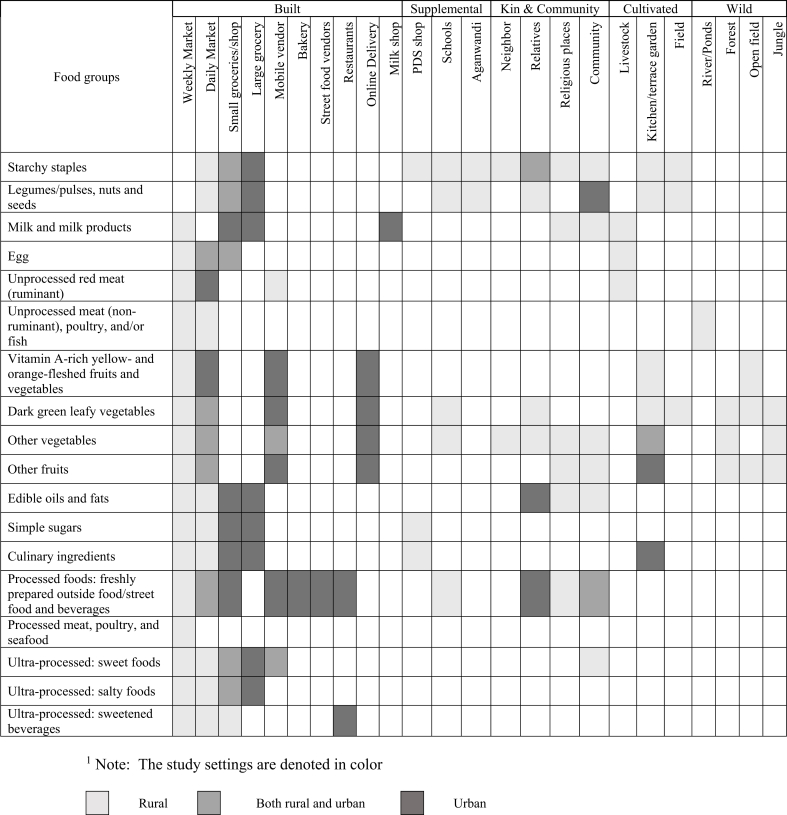
Foods accessed from different types of food environments based on Participatory Mapping focus group discussion piloting in rural and urban India^1^.

### Seasonal Calendar

The Seasonal Calendar provides an overview of the availability of foods across seasons in a given community. Workshop participants in both India and Cambodia viewed the Seasonal Calendar positively overall. In India, participants were split on the length of the tool, with half of the participants indicating that it was appropriate and the other half finding it lengthy; in Cambodia, where the revised version of the tool was piloted, none of the workshop participants rated the length negatively ("disagree" or "strongly disagree").

During the piloting of the tool in rural India, we found that participants seemed restless due to the length of time that it took to complete the discussion. We streamlined the FGD facilitation by asking more general questions like “Which of the listed food items are available throughout the year with little or no variation in availability?” rather than having participants provide a score for each listed item one by one. This reduced the time it took to complete the listing exercise and the FGD more broadly. We also revised the FGD guide to organize it by food groups to decrease the amount of time taken to complete the discussion.

**FIGURE 5:**
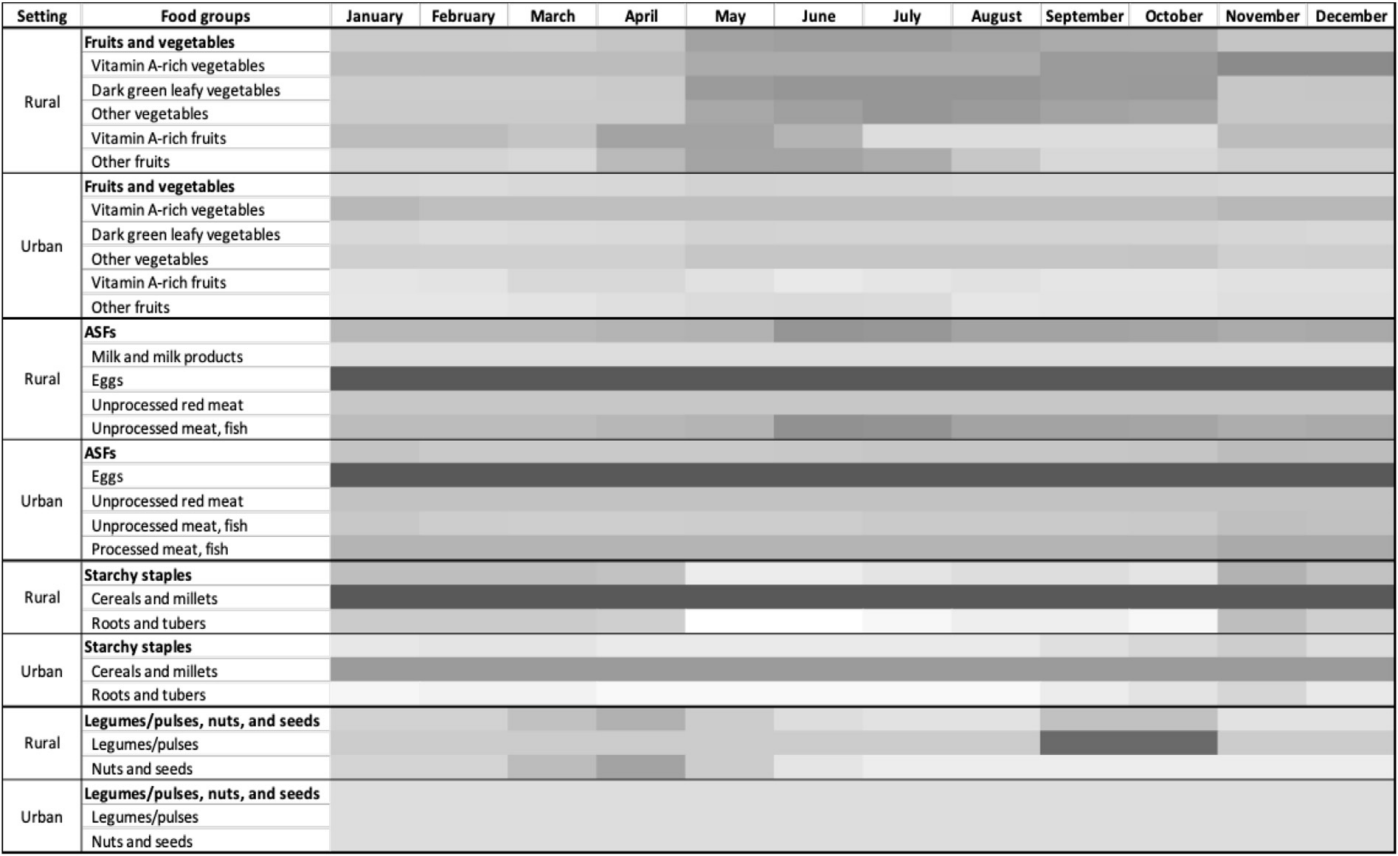
Pilot data of the seasonal availability of foods in rural and urban settings in Cambodia, with perceived availability ranging from no availability (white) to high availability (dark gray). ASF: Animal-source foods.

As part of the analysis of the pilot data, we created heat maps to depict the availability of foods across seasons. We found that there was little variation in the seasonal availability of foods in urban settings, suggesting that the Seasonal Calendar is better suited to rural areas (see Figure 5). Moreover, we found that the diversity of foods in the rural and peri-urban settings was much higher than in the urban settings, which can be attributed to the presence of both wild and cultivated food environments.

### Community Food Environment Mapping

The Community Food Environment Mapping includes 3 tools that together capture vendor types, the food groups they sell and promote, and the GPS coordinates of all food outlets in a given community, in addition to roadside food and/or beverage promotions. Overall, in both the workshops in India and Cambodia, workshop participants viewed the suite of Community Food Environment Mapping tools positively. In India, across all workshop survey questions for the tool, an average of 70% were rated positively, whereas in Cambodia, an average of 93% of responses were rated positively. After receiving feedback during the workshop held in India, we added questions to the promotion section to specify whether promotions are targeting children and to rate how visible promotions are to consumers (e.g., whether they are faded).

**FIGURE 6:**
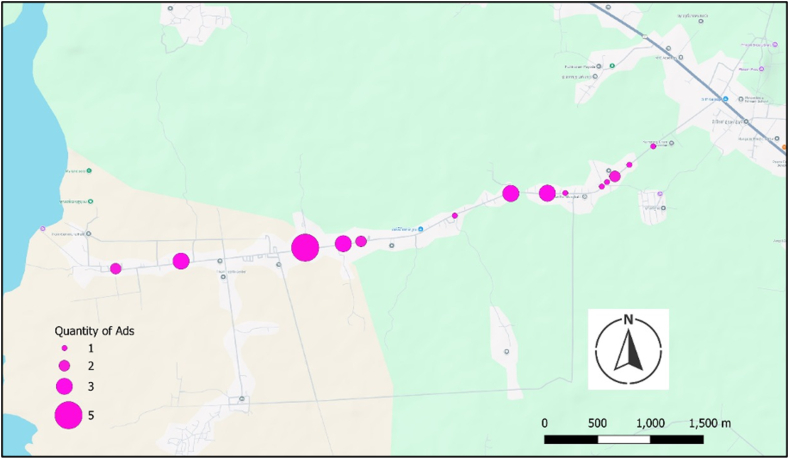
Map of roadside alcohol promotions (the only food and beverage for which roadside promotions were observed) in rural Kampong Siem district, Cambodia.

During the piloting of the tool, the Community Food Environment Mapping Food Outlet Census was relatively quick to complete at each food outlet. On the basis of data from the tablet, the median time that it took to complete the tool in Cambodia was 4 min per food outlet (range from 1 to 12 min). In the piloting, we made small changes such as adding “semi-mobile” as an option under the level of permanence of food outlets/vendors. The Roadside Food and Beverage Promotions module that was part of the Community Food Environment Mapping tool was also relatively quick to administer (median 3 min per promotion in Cambodia based on data from the tablet). Figure 6 provides an example of the number of roadside food and beverage promotions observed along a main road in a rural community in Cambodia.

In analyzing data collected during the pilots, there were stark differences in the number of roadside promotions in India as compared with Cambodia. In the Mandla district in Madhya Pradesh, India, there were no roadside promotions observed in peri-urban and rural areas. Whereas in Cambodia, enumerators observed roadside promotions across every study setting.

Furthermore, in the piloting, the prominence of mobile vendors differed markedly across countries and settings. In Cambodia, mobile vendors were present across the gradient of urbanicity, whereas in India they were much more prominent in urban settings. In particular, in the low-income urban setting in Cambodia, the number of mobile vendors that sold food in the community on the day of the data collection was high (*n* = 78) compared with the rural setting (*n* = 18). This led us to update the instructions to provide better guidance on how the mobile vendor data can be captured when the density of vendors is so high.

### Market Mapping

The Market Mapping tool is an observational checklist that enumerators complete to capture the different vendors in markets as well as the foods that they sell and promote. Several workshop participants in India (36%) indicated they viewed the length of the Market Mapping assessment to be lengthy. To reduce the length of time needed, we removed the initial market transect walk that was conducted to count the total number of vendors before completing the census of all vendors in the market. Respondents in the subsequent workshop in Cambodia responded positively to the tool’s length [no respondents viewed the length of the tool negatively ("disagree" or "strongly disagree"), and only 1 scored its length as neutral].

During the piloting of the tool, the Market Mapping tool was straightforward for enumerators to implement in the field. While conducting the piloting in rural India, the enumerators identified the need to engage a key informant in the market to obtain accurate responses to some of the questions about market properties and infrastructure. This was integrated into the data collection guide and subsequently added to the instructions for the tool. In Cambodia, we further adapted the tool for use in the field. We conducted the urban Market Mapping during very high temperatures, leading us to reconsider the need to conduct a full census of the market vendors. Given the heat stress experienced by enumerators, the finalized tool has an option for conducting a subsample rather than a full census of vendors. This approach was supported by the data, given that we found limited variability in the foods sold by vendor type.

When analyzing the pilot data, the most common vendors in the markets were vegetable vendors in India (47%) and small groceries/kiosks in Cambodia (26%). It was relatively easy for enumerators to classify vendor types based on the majority of foods they sold (60% or more is the guidance provided in the tools). Animal-source foods were 1 exception; in Cambodia, eggs and meats or fish would sometimes be sold in relatively equal amounts, making it difficult to classify the vendors by a “majority” food type. This led us to collapse the animal-source food vendor types in the final version of the tools.

### In-depth Vendor Assessment

The In-depth Vendor Assessment is an observational checklist to be completed by enumerators with a subsample of vendors in communities or markets and provides detailed information related to the specific foods they sell, the use of labeling, vendor hygiene, and food storage, among others. Respondents in both the workshop in India and in Cambodia rated the In-depth Vendor Assessment positively overall; however, some respondents in India (30%) found it to be lengthy. During the workshop, participants suggested adding a question to collect information on whether vendors were registered. We incorporated a question to ascertain whether the vendor was registered and included this in the piloted version; however, registration status was often difficult for enumerators to assess, so this was made optional in the final version of the tool.

During the pilot testing, the In-depth Vendor Assessment took an average of 19 min to complete in Cambodia (range 4–50 min based on the time stamp data from the tablets). In Cambodia, enumerators found it time consuming to conduct the assessment in the large formal supermarkets in the high-income urban setting. In India, the In-depth Vendor Assessment took 15–20 min to complete, and the assessment was conducted without significant challenges even in urban supermarkets. However, in all other vendor types, the assessment was relatively straightforward to implement.

Although other assessments in the Toolbox provide information about the food groups being sold, the In-depth Vendor Assessment provides details about the specific foods within each food group. For example, the tool does not just specify that leafy greens are being sold but specifies the varieties of leafy greens sold. Specifically, in our pilot, the tool captured a wide range of starchy staples being sold in India whereas in Cambodia there were far more varieties of dark leafy green vegetables, but few varieties of starchy staples. The In-depth Vendor Assessment also captures specific data about the use of labeling, how food is stored, vendor hygiene, and sustainability properties.

### Cost of a Healthy Diet

The CoHD data collection protocol contains instructions for collecting item price data, to assess the aspect of costs and affordability in the food environment. Workshop participants in India suggested linking the CoHD protocol with the Community Food Environment Mapping Food Outlet Census tool. As such, we modified the protocol to include additional information about how the food outlet census tool could be used to describe different vendor types and inform price data collection. In Cambodia, workshop participants highlighted the need to include explicit instructions in the protocol to first assess whether using existing data sources might be an option for calculating CoHD, before conducting primary data collection. These instructions were added.

During the pilot data collection, the price data were relatively straightforward to collect based on the protocol. We piloted the tool in 2 ways which were both considered feasible by enumerators: *1*) using a predefined list of foods, and *2*) in an open-ended way using the foods that were available at the markets/vendors. The main challenge identified for collecting the data in an open-ended way was that the enumerators needed to be well trained in how to define the food groups to ensure that the foods were classified in the correct groups. Despite these challenges, both approaches were included in the final data collection tool.

When preparing the data for analysis, there was some difficulty in correctly classifying foods, especially when their local names did not align with their common names or when food groups were misclassified depending on the specific part of the food for which prices were being collected. For example, food price data would be classified differently if an enumerator recorded the price of pumpkin seeds (which would fall under the “Legumes nuts and seeds” food group classification) rather than pumpkin flesh (which would fall under the “Vegetables” food group classification). The need for additional enumerator context highlighted an opportunity to add additional instructions for enumerators and emphasize robust training by the research team. Additionally, it is suggested that enumerators take a photo of the foods for which they are collecting food prices, for later verification. On the basis of these results, the CoHD data collection protocol was included in the Toolbox and linked to the software tools for calculating CoHD on the Food Prices for Nutrition website [26].

### ProDes tool

The ProDes tool uses predetermined criteria to rate the characteristics (e.g., overall desirability, visual appeal, size, etc.) of fruits, vegetables, and animal-source foods based on their quality and desirability. Workshop participants in both settings identified challenges related to the ability to rate the sensory properties of the selected foods, despite establishing predefined standardized criteria to guide enumerator ratings. Workshop participants raised concerns about inconsistent sensory property ratings, given their subjective nature. After the workshop discussions in India, we changed the scoring of ProDes from a score of 0–6 to low, medium, and high quality to increase the consistency of ratings across enumerators. In Cambodia, workshop participants were supportive of the low-, medium-, and high-quality ratings, but they raised questions related to the objectiveness of the tool and whether it was capturing the aspects of quality that influence Cambodian consumers’ food purchasing.

When piloting the updated version of the tool, our team and the enumerators faced several challenges. In India, we were unable to reach consensus for enumerators’ scoring of foods based on their overall desirability, visual desirability, desirability of touch, and desirability of aroma (based on the Standardized Criteria for ProDes Sensory Evaluation table). Given the lack of agreement among the team in terms of how fruits, vegetables, and animal-source foods should be rated, we decided to use the FGDs conducted as part of the Participatory Mapping tool to ask participants about how they determined the quality of different fruits, vegetables, and animal-source foods. We piloted this additional section in the Participatory Mapping discussion in both India and Cambodia.

In analyzing the pilot data, we observed that participants felt that selecting only fruits and vegetables was limiting and that there were disagreements among participants about how to rate the quality of foods (e.g., some viewing insects on food as a positive in Cambodia given that they attributed it to less pesticides, whereas others viewed insects on foods as a negative indicator of quality; in India, the level of ripening of the fruit led to differences in opinions about the quality). There were also elements of perceived quality that were not well-captured in the tool (e.g., where it was produced, how it was produced, when it would be consumed, and its taste). As a participant in the peri-urban setting in Cambodia said: “When we go to the market, we all prefer to eat different fruits” highlighting the difficulty in identifying 5 key fruits that are commonly consumed by all consumers, as is recommended in the tool. In Cambodia, focus group participants described cutting open the fruit to check the quality or tasting the fruit prior to deciding whether to purchase it in addition to using the place of production as a proxy for quality (e.g., domestic fruits from Cambodian provinces being perceived as higher quality than those from Vietnam due to perceived use of chemicals as well as taste properties).

**FIGURE 7:**
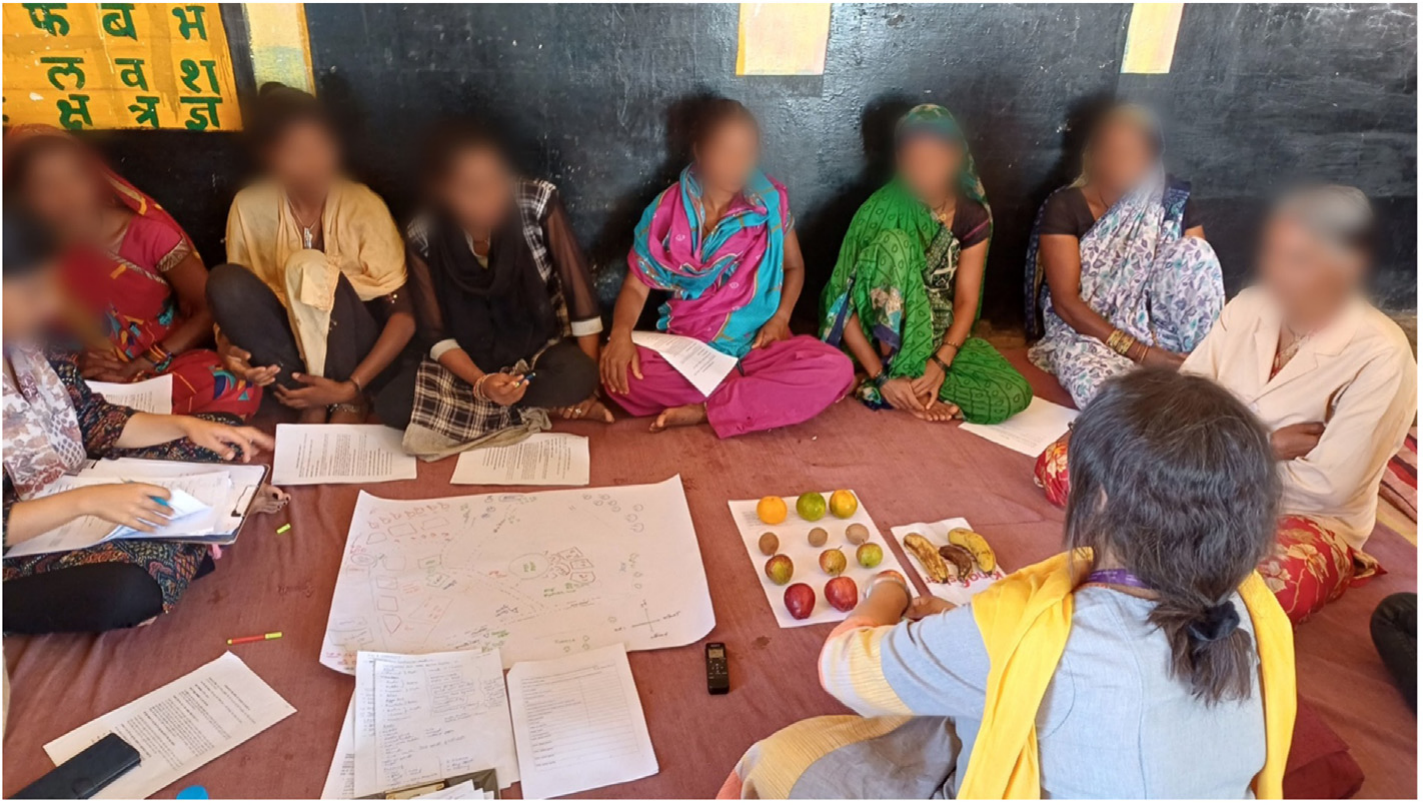
Focus group discussion participants completing the Participatory Mapping exercise and rating fruits as low, medium, or high quality in rural India.

While conducting the FGDs in rural India, participants also disagreed on how to determine whether fruits and vegetables were low-, medium-, or high-quality. Given the iterative nature of the piloting, we decided to pilot another mode of implementing ProDes. We purchased fruits and vegetables from the market and had focus group participants describe and rate their quality (see Figure 7). Participants could not agree on the ratings of the fruits and vegetables. When asked to rate apples, 1 participant said, “one apple is good and other one is little blemished” whereas another participant stated: “I find all the three apples good and acceptable to eat.” Similarly, when discussing grapes, 1 participant stated: “I will buy grapes which are green in color without yellow dots” whereas another participant said: “it does not matter it should taste sweet*.”* In this case, participants weighted desirability differently (e.g., the taste compared with appearance of the foods they purchase), making it difficult to obtain consistent results from the tool.

Finally, a challenge that enumerators faced while piloting the ProDes tool was the ability to find the selected fruits and vegetables in the study settings. Even though fruits and vegetables that were commonly consumed and in season were selected, the availability of produce differed from 1 market to the next. This made it difficult to conduct the assessment on the same fruits and vegetables and to ensure that the predefined criteria for evaluating them were decided upon a priori. Given the difficulties and inconsistencies that we experienced in the piloting, we decided not to include ProDes in the final Toolbox.

## Discussion

This article examines the development, modification, refinement, and implementation of a suite of tools to measure food environments in LMICs. Over the course of 2 and a half years, our team engaged in an iterative process of developing and refining tools, ascertaining feedback on their utility from key food environment experts, and pilot testing them to elicit further information about their feasibility and user acceptance based on enumerator experience. Several iterations were developed throughout this process to inform the final versions of tools that would be most useful for researchers and practitioners and that would be easy to use for enumerators across diverse contexts in LMICs. Apart from the ProDes tool, the tools that we presented at workshops and piloted in India and Cambodia remained in the final Food Environment Toolbox. We anticipate that these tools will be useful to researchers and practitioners working in LMIC settings for measuring food environment dimensions in diverse contexts.

**FIGURE 8:**
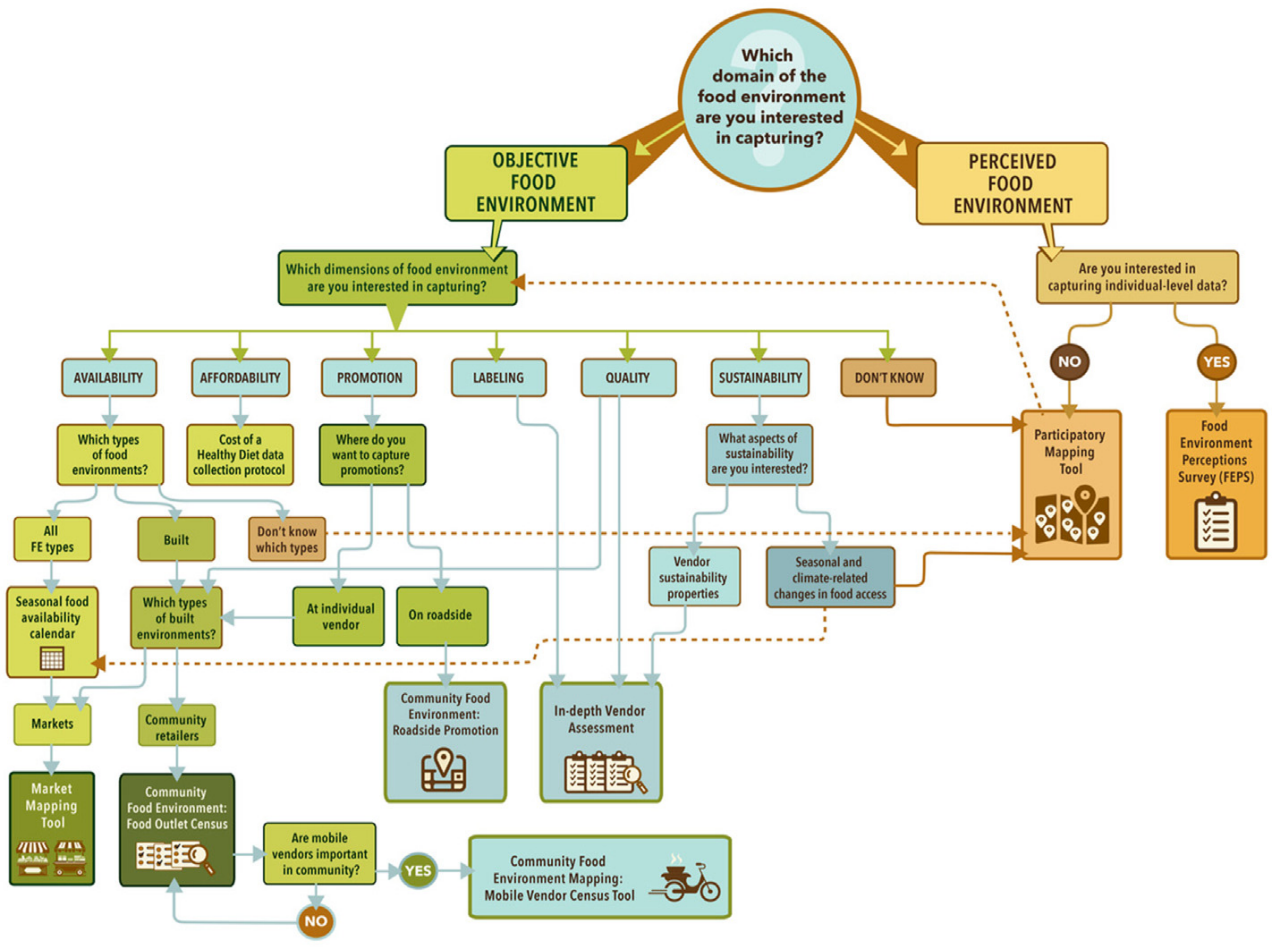
A decision tree for selecting tools from the Food Environment Toolbox.

Overall, the tools were rated positively by experts in workshops in both countries, which is aligned with previous research ascertaining the suitability of versions of some of the Toolbox tools by global food environment experts [24]. While piloting the tools, we found them to be relatively quick and easy to implement in the field. On the basis of the feedback from workshop participants, as well as the piloting of the tools across different settings in both India and Cambodia, we identified which tools might be best suited to different settings and research goals as well as how best to sequence the implementation of the tools. Figure 8 provides a decision tree that can be used to help guide the selection of tools for researchers and practitioners planning to conduct Food Environment Assessments. We found the Participatory Mapping tool to be a relatively low-resource tool and a useful starting point for eliciting information about the types of food environments and foods that the communities’ access, which has also been found in previous food environment research [33,40]. This tool provides a helpful guide for selecting other tools from the Toolbox based on the study context. We found that the Seasonal Calendar may be better suited to rural and peri-urban settings, given the greater diversity of foods found within them, as well as the greater variation over the course of the year. This is perhaps unsurprising given that the methodology was initially developed for rural settings [41]. The CoHD indicator, which includes the cost of each food group recommended for daily consumption, can be used to identify seasonal variation in prices in urban, peri-urban, and rural food environments. Seasonal fluctuations in prices even in urban areas are driven by seasonal food availability in adjacent rural and peri-urban areas, and the use of the Seasonal Calendar and CoHD in tandem may provide important insights. We found the Community Food Environment Mapping Food Outlet Census module to work well across different study settings in India and Cambodia; however, the Roadside Food and Beverage and Mobile Vendor Census modules were more relevant to urban settings in India, while being applicable across all settings in Cambodia. However, as food and beverage advertising continues to expand in LMICs [42], it is likely that this tool will become increasingly relevant across expanded settings. Additional piloting across different country contexts can help to provide additional guidance on where and when to use which tools in the Toolbox.

The Community Food Environment and Market Mapping tools provide a good overview of the foods available within the built food environment. However, the collection of information about the foods that are available within the wild and cultivated environments is only captured in the Seasonal Food Availability Calendar and the Participatory Mapping FGDs. Researchers and practitioners who are aiming to collect quantitative data regarding the availability of foods from wild and cultivated environments and the attributes of food environments may need to use additional tools. For example, the Tool for Agroecology Performance Evaluation, which assesses the agroecological performance of the food systems in terms of parameters such as diversity, cocreation and sharing of knowledge, synergies, efficiency, recycling, resilience, human and social value, culture and food traditions, land and natural resources governance and circular economy [43]. Moreover, capturing the GPS coordinates of food sources beyond the built environment could help to provide a more comprehensive picture of food access within a given community.

### Challenges and limitations

Although the goal of the Food Environment Toolbox was to develop a suite of tools that could assess the different dimensions of the food environment in LMICs, some gaps in the Toolbox remain. One key area that has been identified as a gap in the food environment assessment landscape is tools that measure quality and food safety [32,44]. Although we planned to use the ProDes tool in the Toolbox, as was done in the original USAID Advancing Nutrition Guidelines for Market-based Assessments [25], our piloting experience demonstrated that we were not capturing the dimensions of quality that influenced consumers’ decisions well enough with the tool. It is likely that this tool is more appropriate in high-income settings, where it has been previously shown to work well [45]. The need to design new tools that measure food quality and safety in LMIC contexts has been identified as a food environment assessment gap [46]. The In-depth Vendor Assessment does include questions related to food safety and hygiene practices, based on USAID/GAIN EatSafe Project as part of the observational checklist. In addition, we added a question to the Food Outlet Census and Mobile Vendor Census that asks enumerators to rate the overall quality of food from the perspective of spoilage, damage, and insects on the food. Although the Toolbox has questions related to food safety and vendor hygiene in the In-depth Vendor Assessment as well as an overarching question related to food quality (from the perspective of spoilage, damage, and insects on the foods) in the Food Outlet and Mobile Vendor Censuses, given that consumers often discuss their perceptions about use of chemicals [32,47,48], objective measures of contaminants would complement the tools included in the Toolbox. The Toolbox does not substitute for quantitative objective assessment of hazards that are not necessarily visible, such as testing food samples from vendors for pathogens, aflatoxins, or heavy metals. Identifying field-ready, affordable, and user-friendly food safety assessments for quantitatively measuring contaminants and pathogens would also help complement the tools in the Toolbox [49]. The FEPS can provide insight into how consumers perceive the quality and safety of the foods available within their environments, which could help to identify where more targeted objective assessments of food quality and safety might be warranted.

A key challenge that we faced throughout the tool development was reaching consensus on the food groups to be used in the tools. Although we finalized a list of food groups to be included in the tools, we also indicated that researchers and practitioners will need to make changes to the classifications based on their needs. A key consideration when going through the process of refining and finalizing the food groups was the ease with which enumerators will be able to interpret the groups. Enumerators having difficulty with the diet-related food group classifications have been identified as a challenge in other food environment tool development as well [32,50].

In conclusion, we developed a suite of expert-informed, field-tested tools to measure food environments in LMICs. The tools included in the Toolbox underwent an extensive process of refinement and modification leading to their finalized versions. Overall, the final tools developed as part of the Food Environment Toolbox were relatively easy to implement in the field. Although the tools have been pilot tested in diverse settings in India and Cambodia, we may find ways to refine them further with additional piloting in other settings. Future work will focus on piloting the tools more widely, validating the tools across diverse contexts, and developing innovative data analysis protocols to utilize the information gathered using these tools in a meaningful way.

## Author contributions

The authors’ responsibilities were as follows – SMD, SS, SA, ELF, AH, SG-J: designed research; SMD, SS, NB, NC, PT, NP, TOC, SG-J: conducted data collection; SMD, NC, WS: analyzed data; SMD, NB, WS: drafted the initial manuscript; SS, NC, PT, NP, TOC, SA, ELF, AH, SG-H: provided critical feedback; SMD: primary responsibility for final content; and all authors: read and approved the final manuscript.

## Data availability

Data described in the manuscript will be made available upon request.

## Funding

This work was funded through the Innovative Methods and Metrics for Agriculture and Nutrition Action (IMMANA) program, led by the London School of Hygiene & Tropical Medicine, in partnership with Tufts University and the University of Sheffield. IMMANA is cofunded with UK Aid from the UK government and by the Gates Foundation INV-002962/OPP1211308. Under the grant conditions of the Foundation, a Creative Commons Attribution 4.0 Generic License has already been assigned to the Author Accepted Manuscript version that might arise from this submission.

## Conflict of interest

SMD reports financial support was provided by Innovative Methods and Metrics for Agriculture and Nutrition Actions. If there are other authors, they declare that they have no known competing financial interests or personal relationships that could have appeared to influence the work reported in this article.
