# Supplementary material for: The Food Environment Toolbox: Developing and Piloting a Suite of Tools to Measure Food Environments in Low- and Middle-Income Countries
Source: Curr Dev Nutr. 2025 Apr 16;9(5):107444. doi: 10.1016/j.cdnut.2025.107444 (PMC12147841; doi:10.1016/j.cdnut.2025.107444)
Supplement: multimedia component 1 [file mmc1.docx]

Supplemental Table 1. An overview of the development and refinement of the tools* included in the Food Environment Toolbox

| **Food Environment Toolbox Tool** | **Module or section of tools** | **Existing tools that informed its development** | **Ways in which existing tools informed final versions** |
| --- | --- | --- | --- |
| Participatory mapping | Focus group discussion (FGD) guide | - Larger participatory mapping literature - USAID AN Social Participatory Mapping [1] - Previous studies by members of our team [2-4] | - Adapted the FGD guide to reduce the length of time it takes to complete - Included all different food environment types - Added additional questions related to different types of food environments that are accessed; changes to food environment access over time and across seasons |
| Seasonal Calendar of Food Availability | Focus group discussion (FGD) guide | - Bioversity International Seasonal Food Availability Methodology Guide [5] | - Small changes were made to the wording of questions to streamline the FGD - Added a section to assess changes in the availability of foods over time |
| Food Environment Perceptions Survey (FEPS) | All sections of tool | - Questions from several existing survey instruments were used to inform questions included in survey [6] | - This was a newly developed tool that included compiling a repository of existing instrument items, conducting a Delphi Survey, cognitive testing and piloting. A more detailed description of the survey development is described elsewhere [6] |
| Community Food Environment Mapping tools | Food outlet properties and food availability | - USAID AN Market Mapping Assessment [1] | - The concept of mapping the community food environment was aligned with the USAID AN Market Mapping Assessment - Major changes made included: collecting primary data rather than relying on existing sources; changing food outlet/vendor types; including an observational checklist of different food groups rather than just noting the food outlet/vendor type; including level of permanence of food outlet/vendor; including the form of the food sold (e.g., raw, deep fried, etc.); including a question about overall quality of food - Developed a separate tool specifically for assessing mobile vendors |
|  |  | - Multisectoral Food and Nutrition Security Project (MUSEFO) survey [7] | - Included questions related to the form in which food was sold |
|  |  | - Feed the Future’s EatSafe: Vendor Observational Checklist [8] | - Included a modified version of a question related to damage of foods |
|  | Food and beverage promotions | - Environmental Profile of a Community’s Health (EPOCH) [9] - USAID AN EPOCH Assessment [1] | - Used the EPOCH assessment to guide the collection of data related to food and beverage promotion - Made changes from a standalone tool to incorporate it within the community food environment mapping and in-depth vendor assessment tools to assess food outlet/vendor promotions and added an additional tool to specifically measure roadside promotions - Other changes included: specifying a longer list of food groups for which there were promotions, modifying the promotion types and categories; including questions related to visibility/audibility of promotions and whether the promotions targeted children |
| Market mapping tool | Market properties and infrastructure | - USAID AN Market Mapping Assessment [1] | - Modified the wording of questions originally included in the USAID AN Market Mapping Assessment and added new questions were needed |
|  | Market vendor types and roster | - USAID AN Market Mapping Assessment [1] | - Made changes to the approach of data collection to allow for a sub-sample of vendors to be selected for larger markets. - Other changes included: changing food outlet/vendor types; including an observational checklist of different food groups rather than just noting the food outlet/vendor type; including vendor gender, including the form of the food sold (e.g., raw, deep fried, etc.); including a question about overall quality of food |
|  |  | - Multisectoral Food and Nutrition Security Project (MUSEFO) survey [7] | - Included questions related to the form in which food was sold |
|  |  | - Feed the Future’s EatSafe: Vendor Observational Checklist [8] | - Included a modified version of a question related to damage of foods |
|  | Market promotion | - Environmental Profile of a Community’s Health (EPOCH) [9] - USAID AN EPOCH Assessment [1] | - Used the EPOCH assessment to guide the collection of data related to food and beverage promotion - Other changes included: specifying a longer list of food groups for which there were promotions, modifying the promotion types and categories; including questions related to visibility/audibility of promotions and whether the promotions targeted children |
| In-depth vendor assessment | All sections | | - This was a newly developed tool that included some questions that have been used in other observational checklists |
|  | Storage and Hygiene | - World Food Programme’s Market Functionality Index [10] - Feed the Future’s EatSafe: Vendor Observational Checklist [8] - Multisectoral Food and Nutrition Security Project (MUSEFO) survey [7] | - Included questions related to food storage and vendor hygiene modified from these existing observational checklists |
|  | Vendor Promotion | - Environmental Profile of a Community’s Health (EPOCH) [9] - USAID AN EPOCH Assessment [1] | - Used the EPOCH assessment to guide the collection of data related to food and beverage promotion - Other changes included: specifying a longer list of food groups for which there were promotions, modifying the promotion types and categories; including questions related to visibility/audibility of promotions and whether the promotions targeted children |
| Cost of a Healthy Diet data collection protocol | Data collection instructions | - Food Prices for Nutrition protocols [11] | - Developed protocol for primary data collection of food prices including for collecting prices of food of varying quality, convenience, and sustainability properties |

*Please note that an earlier version of many of these tools was used as part of the [River in Transition Project](https://food.climate.columbia.edu/content/food-flows-project) prior to being modified as part of the Food Environment Toolbox project

**References**

1. USAID Advancing Nutrition. Guidelines for Market-Based Food Environment Assessments. Instruction Manual. Arlington, VA: USAID Advancing Nutrition; 2023.
2. Downs SM, Glass S, Linn KK, Fanzo J. The interface between consumers and their food environment in Myanmar: an exploratory mixed-methods study. Public health nutrition. 2019;22(6):1075-88.
3. Downs SM, Fox EL, Mutuku V, Muindi Z, Fatima T, Pavlovic I, et al. Food Environments and Their Influence on Food Choices: A Case Study in Informal Settlements in Nairobi, Kenya. Nutrients. 2022;14(13):2571.
4. Downs S, Manohar S, Staromiejska W, Keo C, Say S, Chhinh N, et al. Centering context when characterizing food environments: the potential of participatory mapping to inform food environment research. Frontiers in Nutrition. 2024;11:1324102.
5. Lochetti G, Meldrum G, Kennedy G, Termote C. Seasonal food availability calendar for improved diet quality and nutrition: Methodology guide. 2020.
6. Downs S, Staromiejska W, Sok S, Ahmed S, Fox E, Herforth A, et al. The Food Environment Perceptions Survey: development and piloting of a survey instrument to assess consumers' interactions with diverse food environments in low- and middle-income countries. Public Health Nutrition. under review.
7. Deutsche Gesellschaft für Internationale Zusammenarbeit (GIZ). The Multisectoral Food and Nutrition Security (MUSEFO) project Cambodia. 2020.
8. GAIN and USAID. Feed The Future’s EatSafe: Evidence and Action Towards Safe, Nutritious Food [Available from: <https://www.gainhealth.org/impact/programmes/eatsafe>.
9. Chow CK, Lock K, Madhavan M, Corsi DJ, Gilmore AB, Subramanian S, et al. Environmental Profile of a Community's Health (EPOCH): an instrument to measure environmental determinants of cardiovascular health in five countries. PloS one. 2010;5(12):e14294.
10. World Food Programme. Market Functionality Index, Technical Guidance. Rome; 2020.
11. Food Prices for Nutrition. Software Tools for Calculating the Cost of a Healthy Diet, Version 7.0. Published July 2024. Tufts University, Boston (USA) 2024 [Available from: https://sites.tufts.edu/foodpricesfornutrition/tools/.

Supplemental Table 2. Food group classifications

| **Food Groups** | **Examples** |
| --- | --- |
| **Core food groups** | |
| Starchy staples | **Examples:** Barley, bread, breadfruit, brown rice, bulgur, cassava meal, cassava/yucca, corn/maize (dried), flours of the whole grains, fonio, fortified flours, green banana maize flour/meal, millet, noodles, oats, pasta, plantains, popcorn, potato, puffed rice, purple sweet potato, quinoa, rice flakes, rice flour, sorghum, taro, turnip, white rice, white sweet potato, whole grain bread |
| Legumes/pulses | **Examples:** Black eye pea/cowpea, chickpea/garbanzo bean, common black bean, dried fava beans, dried peas, lentils, mung beans, pigeon pea, soybeans, soymilk, tempeh, tofu, white beans |
| Nuts and seeds | **Examples:** Almonds, cashews, chestnuts, flax seeds, flavored nuts and seeds, hazelnuts, macadamia nuts, peanut butter, peanuts, pecans, pine nuts, pistachio, pumpkin seeds, nut and seed butters, sesame seeds, sunflower seeds, tahini, walnut, watermelon seeds |
| Milk, yogurt, and fresh cheese | **Examples:** Camel milk, cottage cheese, curd, evaporated milk (unsweetened condensed milk), fermented milk, fresh whole milk from buffalo, fresh whole milk from cow, fresh whole milk from goat, kefir, local cheese, milk powder, mozzarella cheese, skim or low-fat milk, UHT milk (unflavored and unsweetened), yogurt |
| Eggs | **Examples:** Chicken egg, duck egg, quail egg |
| Red meat | **Examples:** Beef, beef liver or other organ meat, buffalo, goat, lamb, pork, sheep (mutton), veal, venison |
| Poultry, game meat, and insects | **Examples:** Ants, camel, canned poultry, chicken, chicken liver or other organ meat, crickets, duck, flying termites, frog, goose, grasshoppers, guinea fowl, locusts, pigeon, quail, rabbit, rat, snails, spiders, turkey, turtles |
| Fish and seafood | **Examples:** Canned fish, canned sardines, crab, dried fish, dried seafood, eel, fermented fish, fish from sea, freshwater fish, shrimp, small fish, smoked fish, squid, tuna |
| Processed meat | **Definition:** The World Health Organization defines processed meat as “…meat that has been transformed through salting, curing, fermentation, smoking, or other processes to enhance flavour or improve preservation. Most processed meats contain pork or beef, but processed meats may also contain other red meats, poultry, offal, or meat by-products such as blood.”  **Examples:** Bacon, bologna, canned meat, chorizo sausage, cold cuts/luncheon meat, corned beef, dried meat, ham, hot dog, pâté, pepperoni, salami, sausage |
| Vitamin A-rich vegetables | **Examples:** Butternut squash, carrots, dried orange-fleshed vegetables, fermented orange-fleshed vegetables, frozen orange-fleshed vegetables, orange-fleshed squash, pumpkin, red/yellow pepper (sweet), sweet potatoes (orange inside) |
| Dark green leafy vegetables | **Examples:** Amaranth (green), arugula, baobab leaves, bean leaves, bok choy, broccoli, cassava greens, chard, Chinese cabbage, collard greens, cowpea leaves, dried dark leafy greens, eggplant leaves, fermented dark leafy greens, frozen dark leafy greens, grape leaves, hibiscus leaves, jute mallow, kale, lettuce (medium and dark green), moringa greens, mustard greens, pumpkin leaves, sorrel, spinach, sweet potato leaves, taro leaves, water spinach, watercress, wild leaves |
| Other vegetables | **Examples:** African eggplant, beet, bitter gourd, bottle gourd, cabbage, cauliflower, celery, chayote, corn (fresh), cucumber, dried other vegetables, eggplant, fermented other vegetables, frozen other vegetables, green beans, green pepper (capsicum), ivy gourd, lettuce (light green), long beans, mushrooms, okra, radish, tomatoes, zucchini |
| Vitamin A-rich fruits | **Examples:** Apricot, cantaloupe (ripe), dried orange-flesh fruits, fermented orange-fleshed fruit, frozen orange-fleshed fruit, mamey sapote, mango (ripe), papaya (ripe), passion fruit, persimmon |
| Other fruits | **Examples:** Apple, avocado, banana, baobab fruit, berries, cherries, coconut flesh, custard apple (sweetsop), dates, dragon fruit, dried other fruit, fermented other fruit, figs, frozen other fruit, grape fruit, grapes, green mango, green papaya, guava, jackfruit, jujube, lychee, mandarin, mulberries, nectarine, orange, peach, pear, pineapple, plum, pomegranate, pomelo, prickly pear, rambutan, sapota/sapodilla, soursop, star fruit, strawberry, watermelon, wild fruits |
| Fats and oils | **Classification:** Animal fats/oils  **Examples:** butter, ghee, lard/pork fat |
|  | **Classification:** Plant fats/oils  **Examples:** coconut oil, groundnut/peanut oil, other plant-based oils, palm oil, safflower oil, soybean oil, sunflower oil, vegetable oil |
|  | **Classification:** Hydrogenated fats, flavored spreads, etc.  **Examples:** Flavored butter, fortified margarine/vegetable fat, margarine |
| Sweets | **Classification:** Grain-based  **Examples:** baked churros, baked doughnuts, cake, cookies/biscuits, fried dough in syrup, pastries, sweet bread, sweet crepe, wafers |
|  | **Classification:** Other sweets  **Examples:** Candy, chocolate, pudding, rice pudding |
|  | **Classification:** Ice cream  **Examples:** frozen yogurt, gelato, ice cream, ice pops, popsicles, sorbet |
| Sweet drinks | **Classification:** Sweet tea/coffee/cocoa/milk-based drinks  **Examples:** 3-in-1 instant coffee, bubble tea, chocolate drinks, chocolate frappe, coffee frappe, flavoured kefir, flavoured milk drinks, fortified milk drinks, milo/Nesquik, sweetened coffee drinks, sweetened condensed milk, sweetened tea |
|  | **Classification:** Fruit juice, smoothies, and other fruit-based sweet drinks  **Examples:** bissap/hibiscus drink, fresh fruit juices, fresh lemonade, fresh sugarcane juice, fruit flavoured drinks, fruit smoothies, ginger drink |
|  | **Classification:** Sodas, energy drinks, sports drinks, etc.  **Examples:** Carbonated lemonade, carbonated malt drinks, diet soft drinks, energy drinks, soft drinks, sports drinks (drinks with electrolytes) |
| Ultra-processed salty snacks | **Examples:** Chips, potato chips, puffs |
| Ultra-processed ready-to-eat/heat foods | **Examples:** Frozen chicken nuggets and sticks, frozen chips/fritters/wedges, frozen dumplings, frozen meals, frozen meat balls, frozen mozzarella sticks, frozen pizza, frozen sausages, indomie noodles, instant noodles, instant soups, Maggi noodles, packaged breads, packaged cakes/muffins, packaged cookies/biscuits, packaged donuts, packaged sugar-coated cereals, packaged sweet bread |
| Prepared foods by street vendors and restaurants | **Classification:** Deep-fried foods  **Examples:** bean fritters, French fries, fried bananas, fried cassava, fried chicken, fried dough, fried fish, fried plantain, fried pork rinds, fried samosa, fried sweet potato, fritters |
|  | **Classification:** Mixed-dishes, soups, and meals  **Examples:** meat and rice, prepared mixed-dishes served by street vendors |
|  | **Classification:** Prepared salads and other fruit- or vegetable-based dishes  **Examples:** Locally prepared salads |
| Salt, MSG, and salty sauces | **Examples:** Bouillon cubes or powder (i.e., vegetable stock cubes, chicken stock cubes), iodized salt, MSG, other salty sauces and seasonings |
| Simple sugars | **Examples:** Brown sugars, coconut sugar, honey, sugar, syrup |
| Fast food | Foods bought from chain restaurants (i.e., Burger King, Dominos, KFC, McDonald’s, Pizza Hut, Subway, Taco Bell, etc.) such as:  **Examples:** burgers, French fries, fried chicken, pizza |
| **Optional food groups** | |
| Bottled water | **Examples:** Tetra-packed **water** |
| Alcohol | **Examples:** Beer, homebrewed alcohol, liquor, palm wine |
| Fortified mixes | **Examples:** Fortified breakfast mixes, infant mixes |
| Spices, tea, coffee, and condiments | **Examples:** Coconut milk, dried or fresh chilies, fresh or dried spices and herbs, garlic, ginger root, instant coffee powder, roasted cocoa beans/powder, roasted coffee beans/powder, sweetened condensed milk, tea leaves |
| **References** | |
| FAO and FHI 360. 2016. Minimum Dietary Diversity for Women: A Guide for Measurement. Rome: FAO. | |
| WHO International Agency for Research on Cancer (IARC). 2018. Red meat and processed meat. Lyon, France. | |

Supplemental Table 3. Food outlet/vendor classifications used throughout the Food Environment Toolbox

| **Higher level food outlet categorization** | **Secondary level of food outlet categorization** |
| --- | --- |
| Food service (i.e., prepared food) outlet or vendor | Chained fast food restaurant (formal) |
|  | Restaurant/take away (non-chain) shop |
|  | Street food vendor or shop |
|  | Café or coffee vendor or shop |
| Grocery vendor | Supermarket (formal) |
|  | Convenience (formal) |
|  | Small grocery/kiosk |
|  | Large grocery/kiosk |
| Specialty shop or vendor | Bakery |
|  | Meat, fish/seafood, and/or egg shop/vendor |
|  | Vegetable shop/vendor |
|  | Fruit shop/vendor |
|  | Fruit and vegetable shop/vendor |
|  | Milk shop/vendor |
|  | Herb and spice shop/vendor |
|  | Whole dried foods shop/vendor |
|  | Edible oils/fats shop/vendor |
|  | Sweets shop/vendor |
|  | Packaged salty snacks and/or instant noodles shop/vendor |
|  | Sugar-sweetened beverage shop/vendor |
|  | Coffee or tea shop/vendor (does not include prepared coffee or tea) |
| Wholesaler | Mixed goods wholesaler (e.g., grocery) |
|  | Specialty shop wholesaler |
| Supplemental food assistance outlet | Government schemes/public food procurement programs |
|  | Food banks/pantries |
|  | Institutions |
|  | Social services |
