# Supplementary material for: The Food Environment Toolbox: Developing and Piloting a Suite of Tools to Measure Food Environments in Low- and Middle-Income Countries
Source: Curr Dev Nutr. 2025 Apr 16;9(5):107444. doi: 10.1016/j.cdnut.2025.107444 (PMC12147841; doi:10.1016/j.cdnut.2025.107444)
Supplement: multimedia component 2 [file mmc2.pdf]

Appendix A.

Workshop on

The Food Environment (FE) Toolbox: Tools to Measure Natural and Built Food Environments in Low- and Middle-Income Countries (LMIC)
Rutgers School of Public Health, USA
in collaboration with
The George Institute for Global Health, India

Day and Date: Wednesday, 29th November 2023 and Thursday, 30th November 2023
Venue: The Ashtan Sarovar Portico, Green Park, New Delhi - 110016
Timings: 10:00 AM to 05:00 PM

Agenda

Day 1: Session Details		
**Time**	**Theme**	**Session coordinator**
10:00-10:30 am	Opening remarks and introductions	Suparna Ghosh-Jerath
10:30-11:15 am	Introduction to the workshop objectives<br>• Need for Food environment assessment Toolbox for LMICs<br>• List of External and personal food environment tools	Shauna Downs and Suparna Ghosh-Jerath
11:15-11:30 am	Tea break	
11:30-12:00 noon	Participatory mapping of food environment tool	Suparna Ghosh-Jerath
12:00-12:10 pm	Discussion about the tool	All participants
12:10-12:40 pm	Seasonal food availability calendar	Shauna Downs
12:40-12:50 pm	Discussion about the tool	All participants
12:50-1:30 pm	Lunch break	
1:30-2:30 pm	Community food environment mapping tools<br>• Food outlet census<br>• Mobile Vendor census<br>• Roadside food and beverage promotion	Shauna Downs
2:30-3:00 pm	Discussion about the tools	All participants
3:00-3:30 pm	Cost of a Healthy Diet tool	Shauna Downs
3:30-3:50 pm	Open-air market mapping tool	Wiktoria Staromiejska and Shauna Downs
3:50-4:10 pm	Discussion about the Cost of a Healthy Diet and market mapping tools	All participants
4:10-4:20 pm	Tea break	
4:20- 5:00 pm	Day 1 wrap up	Suparna Ghosh-Jerath and Shauna Downs

Day 2: Session Details		
**Time**	**Theme**	**Session coordinator**
10:00-10:15 am	Welcome and Review of Day's Agenda and Objectives	Suparna Ghosh-Jerath
10:15-10:45 am	In-depth Vendor Assessment tool	Shauna Downs
10:45-11:05 am	Discussion about the tool	All participants
11:05-11:15 am	Tea break	
11:15-12:00 noon	Personal food environment assessment tool	Suparna Ghosh-Jerath
12:00-12:30 pm	Discussion about the tool	All participants
12:30-1:00 pm	ProDesirability+	Wiktoria Staromiejska
1:00-1:25 pm	Discussion about the tool	All participants
1:25-1:55 pm	Lunch break	
1:55-4:30 pm	Field testing the tools and discussion of their feasibility	Wiktoria Staromiejska, Hima Bindu Malla and Snigdha Ranjan
4.30-4.50 pm	Workshop survey	All participants
4:50-5:00 pm	Closing remarks and tea	Suparna Ghosh-Jerath and Shauna Downs

Appendix B.

THE FOOD ENVIRONMENT TOOLBOX

Tools to Measure Natural and Built Food Environments in Low- and Middle-Income Countries (LMIC)

DAY 1

Workshop Assessment Survey

Tools to be reviewed: 1. Participatory mapping tool, 2. Seasonal food availability calendar, 3. Community food environment mapping tools (3.1 Food outlet census, 3.2 Mobile Vendor census, 3.3 Roadside food and beverage promotion), 4. Cost of a Healthy Diet tool, 5. Open-Air Market Mapping

Rutgers School of Public Health

The George Institute for Global Health

Feedback Form
Food Environment Toolbox for LMICs

Greetings! You are being invited to complete this survey as a researcher and expert working in the area of food environments, diets, and nutrition. The goal of this feedback survey is to elicit the perspectives of the workshop participants regarding the content, suitability, applicability, and ease of administration of the tools developed for diverse food environment in low- and middle- income countries (LMICs).

In this survey you are being asked to rate each of the tools used in the Food Environment Toolbox based on the criteria below. In addition, please provide any additional feedback on the tool that you think is important to consider before finalizing the tool.

The list of tools on which we seek your valuable feedback are as follows:

Tool no.	Name
1.	Participatory mapping tool
2.	Seasonal food availability calendar
3.	Community food environment mapping tools<br>• Food outlet census<br>• Mobile Vendor census<br>• Roadside food and beverage promotion
4.	Cost of a Healthy Diet tool
5.	Open-Air Market Mapping
6.	In-depth Vendor Assessment tool
7.	Personal food environment assessment tool
8.	ProDesirability+

Tool 1

Participatory Mapping of food environment tool

Content

Does the tool comprehensively elicit information on the following component?

1. Food access points that individuals utilize in their community.

   Adequately covered Somewhat covered Inadequately covered

   Is there any aspect that you think is missing? If so, could you please elaborate upon the specific aspect that can be added in the discussion guide?

2. Kind of markets accessed.

   Adequately covered Somewhat covered Inadequately covered

   Is there any aspect that you think is missing? If so, could you please elaborate upon the specific aspect that can be added in the discussion guide?

3. Identification of the vendor and market types that food price data should be collected from.

   Adequately covered Somewhat covered Inadequately covered

   Is there any aspect that you think is missing? If so, could you please elaborate upon the specific aspect that can be added in the discussion guide?

4. Kind of natural food environment accessed.

   Adequately covered Somewhat covered Inadequately covered

   Is there any aspect that you think is missing? If so, could you please elaborate upon the specific aspect that can be added in the discussion guide?

5. Key foods that should be included in the Participatory Mapping Tool and their corresponding sensory property attributes.

   Adequately covered   Somewhat covered   Inadequately covered

   Is there any aspect that you think is missing? If so, could you please elaborate upon the specific aspect that can be added in the discussion guide?

6. Do you agree with the current sequence of questions where built food environment questions are asked first and then the natural food environment ones?
   Yes
   No

   If not, why?

Face validity

Please review all the FGD guide question and assess the following.

7. Comprehension: Please tell us if it is easy for the FGD participants to understand and follow the question where we are asking for reasons for accessing least amount of food from a particular access point?

   Easy   Somewhat difficult   Difficult

8. Can you tell us in your own words, what are we trying to find out from these questions?

9. Recall: Please tell us if it will be easy for the FGD participants to collectively recollect all the food items accessed from one access point.

   Easy   Somewhat difficult   Difficult

10. Judgement: Please tell us if it will be easy for the FGD participants to collectively decide the list of food items accessed from one access point.

    Easy   Somewhat difficult   Difficult

Overall assessment

11. How do you find the overall presentation of the tool?

    Well-presented   Somewhat satisfactory   Unsatisfactory

12. Do you think the language of the tool easily comprehensible?

    Easy   Somewhat difficult   Difficult

13. What do you think about the length of the tool?

    Short   Appropriate   Somewhat lengthy   Very lengthy

14. This tool can be easily used in the field in LMIC settings.

    Agree   Somewhat agree   Disagree

15. The data obtained from the tool is relevant to the LMIC populations.

    Agree   Somewhat agree   Disagree

16. The relationship between the tool and diets is:

    Definite   Probable   Speculative

Tool 2

Seasonal food availability calendar tool

Content

Availability

Does the tool comprehensively elicit information on the following component?

1. Food groups for which we are assessing the availability.

   Adequately covered   Somewhat covered   Inadequately covered

   Is there any food group that you think is missing, needs re-grouping or is not applicable? If so, could you please elaborate?

2. The type of seasons covered in the tool

   Adequately covered   Somewhat covered   Inadequately covered

   Please share your opinion, in case you feel that the seasonality component needs additions, re-categorization, etc.

Characteristics of food items for verifying species identification

3. In order to identify an unknown species (Section D, part 3), the questions elicit key information on the characteristics of food items

   Adequately covered   Somewhat covered   Inadequately covered

   Is there any aspect that you think is missing/ not applicable? If so, could you please elaborate?

Sources from where food is accessed

4. Sources from where different food categories are accessed in the community /village

   Adequately covered   Somewhat covered   Inadequately covered

Is there any source that you think is missing/ can be re-categorized/ not applicable? If so, could you please elaborate?

Scoring of seasonal availability

5. Is the scoring system on the seasonal availability clearly explained?

   Yes
   No

   If no, please suggest how it can be made clearer?

Sustainability dimensions

6. The sustainability component elicited as foods consumed historically, food for which consumption declined etc are comprehensively covered

   Agree Somewhat agree Disagree

   Is there any aspect that you think is missing or not applicable? If so, could you please elaborate?

Face validity

Please review all the FGD guide questions and assess the following.

7. Comprehension: Please tell us if it is easy for the FGD participants to understand and follow the question where we are asking about the foods, or varieties of foods, that were being historically consumed in their community that are now unavailable.

   Easy Somewhat difficult Difficult

   Can you tell us in your own words, what are we trying to find out from this question?

8. Recall: Please tell us if it will be easy for the FGD participants to collectively recollect and inform about the food items whose availability has declined in years.

   Easy Somewhat difficult Difficult

9. Judgement: Please tell us if it will be easy for the FGD participants to collectively decide on the degree of availability for each food item in each month of the year.

   Easy Somewhat difficult Difficult

Overall assessment

10. How do you find the overall presentation of the tool?

    Well-presented Somewhat satisfactory Unsatisfactory

11. Do you think the language of the tool is easily comprehensible?

    Easy Somewhat difficult Difficult

12. What do you think about the length of the tool?

    Short Appropriate Somewhat lengthy Very lengthy

13. This tool can be easily used in the field in LMIC settings.

    Agree Somewhat agree Disagree

14. The data obtained from the tool is relevant to the LMIC populations.

    Agree Somewhat agree Disagree

15. The relationship between the tool and diets is:

    Definite Probable Speculative

Tool 3

Community Food Environment Mapping
Tool 1. Food outlet census
Tool 2. Mobile vendor census
Tool 3. Roadside food and beverage promotion

Content of Community Food Environment Mapping Tools overall

1. Is the purpose of this set of tools clear for its applicability in FE assessment in LMIC?

   Adequately covered Somewhat covered Inadequately covered

2. Do you think that the set of tools cover all the relevant dimensions that are required for mapping community food environments?

   Adequately covered Somewhat covered Inadequately covered

3. Is there a need for more clarity, if so, please suggest how can we make it better?

Food groups used in community food environment mapping tools
Please review the food group categories used in the community food environment mapping tools and answer the following:

4. Does the tool capture all the food groups comprehensively?

   Adequately covered Somewhat covered Inadequately covered

5. Are the food categories used in the tool appropriate for categorizing the foods sold in LMICs?

   Yes
   No

6. Is there any food group missing and/or there is a need for re-categorized and/or any category that might be redundant/not applicable? If so, could you please elaborate upon your observations?

Food and beverage promotions used in community food environment mapping tools

Please review the food and beverage promotion categories used in the community food environment mapping tools:

7. Does the set of tools adequately capture food promotion aspect of community food environments in LMICs?

   Agree Somewhat agree Disagree

   Is there any aspect that you think is missing? If so, could you please elaborate upon the specific aspect that can be added in this section?

8. Do you think any of the sections under food promotion is not applicable/ redundant to LMICs?

   Yes
   No

9. If yes, please elaborate with reasons?

Tool specific questions

The following sections ask specific questions about each of the tools included in the Community Food Environment Mapping tools.

<u>Tool 1. Community Food Environment Mapping Tools: Food Outlet Census</u>

Food outlet properties and food availability: Food outlet types

Please review the food outlet types and answer the following:

10. Does the tool capture all the food outlet types comprehensively?

    Adequately covered Somewhat covered Inadequately covered

11. Are the food outlet types relevant to LMICs?
    Yes
    No

12. Is there any food outlet type missing and/or there is a need for re-categorized and/or any category that might be redundant/not applicable? If so, could you please elaborate upon your observations?

Overall assessment of food outlet census tool

13. How do you find the overall presentation of the tool?

    Well-presented   Somewhat satisfactory   Unsatisfactory

14. Do you think the language of the tool easily comprehensible by the field worker?

    Easy   Somewhat difficult   Difficult

15. What do you think about the length of the tool?

    Short   Appropriate   Somewhat lengthy   Very lengthy

16. This tool can be easily used in the field in LMIC settings.

    Agree   Somewhat agree   Disagree

17. The data obtained from the tool is relevant to the LMIC populations.

    Agree   Somewhat agree   Disagree

18. The relationship between the tool and diets is:
    Definite   Probable   Speculative

    Please provide any other feedback you have on this tool.

<u>Tool 2. Community Food Environment Mapping Tools: Mobile Vendor Census</u>

Food outlet properties and food availability: Mobile vendor types

Please review the mobile vendor types and answer the following:

19. Does the tool capture all the mobile vendor types comprehensively?

    Adequately covered   Somewhat covered   Inadequately covered

20. Are the mobile vendor types relevant to LMICs?

    Yes
    No

21. Is there any mobile vendor type missing and/or there is a need for re-categorized and/or any category that might be redundant/not applicable? If so, could you please elaborate upon your observations?

Overall assessment of mobile vendor census tool

22. How do you find the overall presentation of the tool?

    Well-presented   Somewhat satisfactory   Unsatisfactory

23. Do you think the language of the tool easily comprehensible by the field worker?

    Easy   Somewhat difficult   Difficult

24. What do you think about the length of the tool?

    Short   Appropriate   Somewhat lengthy   Very lengthy

25. This tool can be easily used in the field in LMIC settings.

    Agree   Somewhat agree   Disagree

26. The data obtained from the tool is relevant to the LMIC populations.

    Agree   Somewhat agree   Disagree

27. The relationship between the tool and diets is:

    Definite   Probable   Speculative

    Please provide any other feedback you have on this tool.

<u>Tool 3. Community Food Environment Mapping Tools: Roadside food and beverage promotion tool</u>

Overall assessment of the roadside food and beverage promotion tool

28. How do you find the overall presentation of the tool?

    Well-presented   Somewhat satisfactory   Unsatisfactory

29. Do you think the language of the tool easily comprehensible by the field worker?

    Easy   Somewhat difficult   Difficult

30. What do you think about the length of the tool?

    Short   Appropriate   Somewhat lengthy   Very lengthy

31. This tool can be easily used in the field in LMIC settings.

    Agree   Somewhat agree   Disagree

32. The data obtained from the tool is relevant to the LMIC populations.

Agree Somewhat agree Disagree

33. The relationship between the tool and diets is:

Definite Probable Speculative

Please provide any other feedback you have on this tool.

Tool 4

Cost of a Healthy Diet data collection protocol

Content of CoHD data collection protocol

1. Is the purpose of the tool clear in terms of its applicability in FE assessment in LMIC?

   Adequately covered   Somewhat covered   Inadequately covered

2. Do you think that the tool covers all the relevant information that is required for collecting price data in LMICs?

   Adequately covered   Somewhat covered   Inadequately covered

3. Is there a need for more clarity, if so, please suggest how can we make it better?

4. Do you think the tool covers all the necessary information to enable the collection of price data in LMICs?

   Adequately covered   Somewhat covered   Inadequately covered

5. Is there a need for more clarity, if so, please suggest how can we make it better?

6. Do you think the tool covers all the necessary information to enable the collection of variations in price data by quality, convenience, or sustainability characteristics in LMICs?

   Adequately covered   Somewhat covered   Inadequately covered

7. Is there a need for more clarity, if so, please suggest how can we make it better?

Overall assessment of the CoHD data collection protocol

8. How do you find the overall presentation of the tool?

   Well-presented   Somewhat satisfactory   Unsatisfactory

9. Do you think the language of the tool easily comprehensible by the field worker?

   Easy   Somewhat difficult   Difficult

10. What do you think about the length of the tool?

    Short   Appropriate   Somewhat lengthy   Very lengthy

11. This tool can be easily used in the field in LMIC settings

    Agree   Somewhat agree   Disagree

12. The data obtained from the tool is relevant to the LMIC populations.

    Agree   Somewhat agree   Disagree

13. The relationship between the tool and diets is:

    Definite   Probable   Speculative

    Please provide any other feedback you have on this tool.

Tool 5

Open-air market mapping tool

Content of Market Mapping tool

1. Is the purpose of the tool clear in terms of its applicability in FE assessment in LMIC?

   Adequately covered   Somewhat covered   Inadequately covered

2. Do you think that the tool covers all the relevant dimensions that are required for mapping markets in LMICs?

   Adequately covered   Somewhat covered   Inadequately covered

3. Is there a need for more clarity, if so, please suggest how can we make it better?

Food groups used in community food environment mapping tools

Please review the food group categories used in the community food environment mapping tools and answer the following

4. Does the tool capture all the food groups comprehensively?

   Adequately covered   Somewhat covered   Inadequately covered

5. Are the food categories used in the tool appropriate for categorizing the foods sold in LMICs?

   Yes
   No

6. Is there any food group missing and/or there is a need for re-categorized and/or any category that might be redundant/not applicable? If so, could you please elaborate upon your observations?

Food and beverage promotions used in community food environment mapping tools

Please review the food and beverage promotion categories used in the community food environment mapping tools.

7. Does the set of tools adequately capture food promotion aspect of community food environments in LMICs?

   Adequately covered   Somewhat covered   Inadequately covered

8. Is there any aspect that you think is missing? If so, could you please elaborate upon the specific aspect that can be added in this section?

9. Do you think any of the sections under food promotion is not applicable/ redundant to LMICs?

   Yes
   No

10. If yes, please elaborate with reasons?

Module 1. Market properties and infrastructure

Please review the market properties and infrastructure section of the market mapping tool and answer the following questions:

11. Does the tool capture all the important market property and infrastructure considerations for LMICs comprehensively?

    Adequately covered   Somewhat covered   Inadequately covered

12. Are the market property and infrastructure questions relevant to LMICs?

    Yes
    No

13. Is there any market property and infrastructure consideration missing and/or there is a need for re-framing questions, removing questions that are redundant or unnecessary, etc.? If so, could you please elaborate upon your observations?

14. Does the tool capture all the important market types for LMICs comprehensively?

    Adequately covered   Somewhat covered   Inadequately covered

15. Is there any market type missing and /or there is a need for re-categorized and /or any category that might be redundant/not applicable? If so, could you please elaborate upon your observations?

Module 2. Market vendor roster

16. Do you think it's necessary to count the total number of vendors in the market?

    Yes
    No

Please review the market vendor types and answer the following:

17. Does the tool capture all the market vendor types comprehensively?

    Adequately covered   Somewhat covered   Inadequately covered

18. Are the market vendor types relevant to LMICs?

    Yes
    No

19. Is there any market vendor type missing and/or there is a need for re-categorized and/or any category that might be redundant/not applicable? If so, could you please elaborate upon your observations?

Overall assessment of the market mapping tool

20. How do you find the overall presentation of the tool?

    Well-presented   Somewhat satisfactory   Unsatisfactory

21. Do you think the language of the tool easily comprehensible by the field worker?

    Easy   Somewhat difficult   Difficult

22. What do you think about the length of the tool?
    Short   Appropriate   Somewhat lengthy   Very lengthy

23. This tool can be easily used in the field in LMIC settings

    Agree   Somewhat agree   Disagree

24. The data obtained from the tool is relevant to the LMIC populations

    Agree   Somewhat agree   Disagree

25. The relationship between the tool and diets is:

Definite Probable Speculative

Please provide any other feedback you have on this tool.

Tool 6

In-depth Vendor Assessment tool

Content

1. Is the purpose of this tool clear for its applicability in FE assessment in LMIC?

   Adequately covered   Somewhat covered   Inadequately covered

2. Do you think that the tool covers all the relevant dimensions that are required for assessing in-depth vendor assessment?

   Adequately covered   Somewhat covered   Inadequately covered

3. Is there a need for more clarity, if so, please suggest how can we make it better?

Availability

Please review the food group categories and answer the following:

4. Does the tool capture all the food groups comprehensively?

   Adequately covered   Somewhat covered   Inadequately covered

5. Are the food categories used in the tool appropriate for the food categories relevant to LMIC?

   Yes
   No

6. Is there any food group missing and/or there is a need for re-categorized and/or any category that might be redundant/not applicable? If so, could you please elaborate upon your observations?

7. The vendor types under market and community in LMIC context are:

   Adequately covered   Somewhat covered   Inadequately covered

8. Is there any vendor type missing and /or there is a need for re-categorized and /or any category that might be redundant/not applicable? If so, could you please elaborate upon your observations?

Label

Does the tool comprehensively elicit information on the

9. Labelling of different food groups in the LMIC context

   Adequately covered Somewhat covered Inadequately covered

10. Is there any aspect that you think is missing? If so, could you please elaborate upon the specific aspect that can be added in this section?

11. Do you think any of the sections under labelling is not applicable/ redundant to food products in LMICs?

    Yes
    No

12. If yes, please elaborate with reasons?

Sustainability

Does the tool comprehensively elicit information on the

13. Sustainability aspect and related measures taken for different foods sold in the LMIC

    Adequately covered Somewhat covered Inadequately covered

14. Is there any aspect that you think is missing? If so, could you please elaborate upon the specific aspect that can be added in this section?

15. Do you think any of the sections under sustainability is not applicable/ redundant to LMIC context?

    Yes
    No

16. If yes, please elaborate with reasons?

Storage

Does the tool comprehensively elicit information on the

17. Storage of different foods by the vendors in LMIC context

    Adequately covered Somewhat covered Inadequately covered

18. Is there any aspect that you think is missing? If so, could you please elaborate upon the specific aspect that can be added in this section?

19. Do you think any of the sections under food storage is not applicable/ redundant to LMIC context?

    Yes
    No

20. If yes, please elaborate with reasons?

Hygiene

Does the tool comprehensively elicit information on the

21. Hygiene and food safety principles followed by vendors in LMIC context?

    Adequately covered Somewhat covered Inadequately covered

22. Is there any aspect that you think is missing? If so, could you please elaborate upon the specific aspect that can be added in this section?

23. Do you think any of the sections under Hygiene is not applicable/ redundant to LMIC context?

    Yes
    No

24. If yes, please elaborate with reasons?

Food Promotion

Does the tool comprehensively elicit information on the

25. Food promotion aspect of different vendor types in LMICS

    Adequately covered Somewhat covered Inadequately covered

26. Is there any aspect that you think is missing? If so, could you please elaborate upon the specific aspect that can be added in this section?

27. Do you think any of the sections under food promotion is not applicable/ redundant to food categories and vendor types in LMICs

    Yes
    No

28. If yes, please elaborate with reasons?

Overall assessment

29. How do you find the overall presentation of the tool?

    Well-presented Somewhat satisfactory Unsatisfactory

30. Do you think the language of the tool easily comprehensible by the field worker?

    Easy Somewhat difficult Difficult

31. What do you think about the length of the tool?
    Short Appropriate Somewhat lengthy Very lengthy

32. This tool can be easily used in the field in LMIC settings.

    Agree Somewhat agree Disagree

33. The data obtained from the tool is relevant to the LMIC populations

    Agree Somewhat agree Disagree

34. The relationship between the tool and diets is:
    Definite Probable Speculative

Tool 7

Personal Food environment survey tool

Content

1. Do you think that the tool covers all the relevant dimensions of personal food environment assessment?

   Adequately covered Somewhat covered Inadequately covered

2. Which aspect is missing, could you please elaborate upon on that?

Accessibility

Does the tool comprehensively elicit information on the following dimensions?

3. Accessibility to different kind of food environment by the respondent

   Adequately covered Somewhat covered Inadequately covered

4. Is there any aspect that you think is missing? If so, could you please elaborate upon the specific aspect that can be added in this section?

5. Do you think any of the sections under accessibility section is not applicable/ redundant to food environment categories in LMIC?

   Yes
   No

6. If yes, please elaborate with reasons?

7. The examples given under different access points in the accessibility section are

   Adequately covered Somewhat covered Inadequately covered

8. Does any food access point need more examples? If so, could you please elaborate upon the specific section with examples?

9. Foods accessed from different access points

   Adequately covered   Somewhat covered   Inadequately covered

10. Is there any aspect that you think is missing? If so, could you please elaborate upon the specific aspect?

11. Would you like to suggest a different categorization of food groups for this tool?

Affordability

Does the tool comprehensively elicit information on the

12. Affordability aspects related to different food groups

    Adequately covered   Somewhat covered   Inadequately covered

13. Is there any aspect that you think is missing? If so, could you please elaborate upon the specific aspect that can be added in this section?

14. Do you think any of the sections under affordability is not applicable/ redundant to food environment categories in LMIC?

    Yes
    No

15. If yes, please elaborate with reasons?

Does the tool comprehensively elicit information on the

16. Convenience aspects related to different food groups

    Adequately covered   Somewhat covered   Inadequately covered

17. Is there any aspect that you think is missing? If so, could you please elaborate upon the specific aspect that can be added in this section?

18. Do you think any of the sections under convenience is not applicable/ redundant to food environment categories in LMIC?

Yes
No

19. If yes, please elaborate with reasons?

Does the tool comprehensively elicit information on the

20. Food quality and safety aspects related to different food groups

Adequately covered Somewhat covered Inadequately covered

21. Is there any aspect that you think is missing? If so, could you please elaborate upon the specific aspect that can be added in this section?

22. Do you think any of the sections under food quality and safety is not applicable/ redundant to food environment categories in LMIC

Yes
No

23. If yes, please elaborate with reasons?

Food Promotion and Labelling

Does the tool comprehensively elicit information on the

24. Food promotion and labelling aspects in food environment of LMICS

Adequately covered Somewhat covered Inadequately covered

25. Is there any aspect that you think is missing? If so, could you please elaborate upon the specific aspect that can be added in this section?

26. Do you think any of the sections under food promotion and labelling is not applicable/ redundant to food environment categories in LMIC?

Yes No

27. If yes, please elaborate with reasons?

Sustainability

Does the tool comprehensively elicit information on the

28. Sustainability aspect of food environment of LMICS

Adequately covered Somewhat covered Inadequately covered

29. Is there any aspect that you think is missing? If so, could you please elaborate upon the specific aspect that can be added in this section?

30. Do you think any of the sections under sustainability is not applicable/ redundant to food environment categories in LMIC?

Yes
No

31. If yes, please elaborate with reasons?

Food sovereignty

Does the tool comprehensively elicit information on the

32. Food sovereignty aspect of food environment of LMIC

    Adequately covered Somewhat covered Inadequately covered

33. Is there any aspect that you think is missing? If so, could you please elaborate upon the specific aspect that can be added in this section?

34. Do you think any of the sections under food sovereignty is not applicable/ redundant to food environment categories in L

    Yes
    No

35. If yes, please elaborate with reasons?

Face validity

Please review all the question and assess the following.

36. Comprehension: Please tell us if it is easy for respondents to understand and follow the question where we are asking about convenience attributes of different foods (section 3.1)?

    Easy Somewhat difficult Difficult

37. Can you tell us in your own words, what are we trying to find out from these questions?

38. Recall: Please tell us if it will be easy for respondents to respond to questions such as what they or the person who mostly prepares food at home does to overcome time constraint in food preparation

    Easy Somewhat difficult Difficult

39. Recall: Please tell us if it is easy for respondents to respond to questions on recalling different kinds of food labels

    Easy Somewhat difficult Difficult

40. Judgement: Please tell us if it will be easy for respondents to respond to questions on food advertisements and the attributes that make them buy food

    Easy   Somewhat difficult   Difficult

Overall assessment

41. How do you find the overall presentation of the tool?

    Well-presented   Somewhat satisfactory   Unsatisfactory

42. Do you think the language of the tool easily comprehensible?

    Easy   Somewhat difficult   Difficult

43. What do you think about the length of the tool?

    Short   Appropriate   Somewhat lengthy   Very lengthy

44. This tool can be easily used in the field in LMIC settings

    Agree   Somewhat agree   Disagree

45. The data obtained from the tool is relevant to the LMIC populations

    Agree   Somewhat agree   Disagree

46. The relationship between the tool and diets is:

    Definite   Probable   Speculative

Tool 8
ProDesirability+ tool

Content of ProDesirability+ tool

1. Is the purpose of the tool clear in terms of its applicability in FE assessment in LMIC?

   Adequately covered   Somewhat covered   Inadequately covered

2. Do you think that the tool covers all the relevant dimensions that are required for assessing the quality and/or desirability of fresh foods in LMICs?

   Adequately covered   Somewhat covered   Inadequately covered

3. Is there a need for more clarity, if so, please suggest how can we make it better?

Selection of foods to be assessed using the tool

4. Do you think selecting a small number (5 or more) of key fruits, vegetables, and animal-source foods to be assessed using the ProDesirability+ tool is appropriate for LMIC contexts?

   Yes
   No

5. Please elaborate upon your observation?

6. Do you think including animal-source foods in the sample of foods to be assessed using the ProDesirability+ tool is appropriate for LMIC contexts?

   Yes
   No

7. Please elaborate upon your observation?

Different sensory properties assessed by the ProDes+ tool

8. Do you think that the tool covers all the relevant sensory properties (overall desirability, visual desirability, desirability of touch, desirability of aroma, desirability of size) that are required for assessing the quality and/or desirability of fresh foods in LMICs?

   Adequately covered Somewhat covered Inadequately covered

9. Is there a need for more clarity, if so, please suggest how can we make it better?

Standardized Criteria for ProDes+ Sensory Evaluation of fruits, vegetables, and animal-source foods

Please review the predetermined criteria for sensory properties section of the ProDesirability+ tool and answer the following questions

10. Does the approach to selecting the criteria for low, medium, and high-quality fresh foods seem appropriate for LMIC contexts?

    Yes
    No

    Please elaborate upon your observation?

Sensory property ratings for fresh foods included in the assessment

11. Do you think rating key fruits, vegetables, and animal-source as low-, medium-, or high-quality will be feasible for data collectors?

    Yes
    No

    Please elaborate upon your observation?

Overall assessment of the ProDesirability+ tool

12. How do you find the overall presentation of the tool?

    Well-presented   Somewhat satisfactory   Unsatisfactory

13. Do you think the language of the tool easily comprehensible by the field worker?

    Easy   Somewhat difficult   Difficult

14. What do you think about the length of the tool?

    Short   Appropriate   Somewhat lengthy   Very lengthy

15. This tool can be easily used in the field in LMIC settings.

    Agree   Somewhat agree   Disagree

16. The data obtained from the tool is relevant to the LMIC populations

    Agree   Somewhat agree   Disagree

17. The relationship between the tool and diets is:

    Definite   Probable   Speculative

Please provide any other feedback you have on this tool.

Appendix C.

Workshop
on
The Food Environment (FE) Toolbox: Tools to Measure Natural and Built Food Environments in Low- and Middle-Income Countries (LMIC)

Date: Friday, 29th March 2024 from 8:30AM to 4:30PM
Venue: The Cambodia Korean Cooperation, Royal University of Phnom Penh (RUPP), Russian Federation Boulevard, Toul Kork, Phnom Penh, Cambodia
Organizer: Rutgers School of Public Health, USA and the Royal University of Phnom Penh, Cambodia
Contact: Dr. Serey Sok, Tel: 085 705 719/ Tel: 016521 574

Workshop Agenda

Time	Theme	Session coordinator
8:00AM—8:30AM	Registration	Ms. Leakhena Thoeun and Mr. Phal Vipharo
8:30AM—9:00AM	Opening remarks and introductions<br>Dr. Serey Sok, Vice-rector (in charge of research), Royal University of Phnom Penh<br><br>[Group Photo]	Ms. Leakhena Thoeun
9:00AM—9:30AM	Introduction to the workshop objectives<br>• Need for food environment assessment Toolbox for LMICs<br>• List of External and personal food environment tools	Dr. Shauna Downs
9:30AM—10:00AM	Overview of tools: **Participatory mapping** and **Community Food Environment Mapping**	Dr. Serey Sok, Dr. Shauna Downs and Dr. Nyda Chhinh
10:00AM—10:20AM	Coffee break	Mr. Phal Vipharo and Mr. Sam Ath Boramey
10:20AM—11:00AM	Overview of tools: **Market mapping** and **In-depth vendor assessment**	Dr. Shauna Downs and Dr. Nyda
11:00AM—11:40AM	**Group Discussion**<br>Group 1: Participatory Mapping<br>Group 2: Community Food Environment Mapping<br>Group 3: Market Mapping<br>Group 4: In-depth vendor assessment<br><br>**Instructions**: Each group will be asked to go over their assigned tool in detail and provide feedback on how it can be improved and any corrections needed.	Dr. Shauna Downs<br>Dr. Nyda Chhinh<br>Mr. Thou Ponlue<br>Ms. Leakhena Thoeun<br>Mr. Nayelin Phorn<br>Mr. Hang Sovichea
11.40AM—12:00PM	**Group Presentation (5 minute each)**<br>Group 1: Participatory Mapping<br>Group 2: Community Food Environment Mapping	Dr. Shauna Downs and Dr. Nyda Chhinh

	Group 3: Market Mapping<br>Group 4: In-depth vendor assessment<br><br>Questions and answers	
12:00PM—01:00PM	Break Lunch	Mr. Vipharo Phal and Mr. Sam Ath Boramey
01:00PM—01:30PM	Overview of tool: **Personal Food Environment Survey Instrument**	Dr. Shauna Downs
01:30PM—02:00PM	Overview of tools: **Seasonal food availability calendar** and **Cost of a Healthy Diet data collection**	Mr. Chen Tepsam Ol, Dr. Serey Sok, Dr. Shauna Downs, and Ms. Leakhena Thoeun
02:00PM—02:30PM	Coffee break	Mr. Vipharo Phal and Mr. Sam Ath Boramey
02:30PM—02:45PM	Overview of tools: **ProDesirability+**	Dr. Shauna Downs, Mr. Nayelin Phorn, Mr. Thou Ponlue
02:45PM—3:45PM	**Group Discussion**<br>Group 1: Personal Food Environment (sections 1-3)<br>Group 2: Personal Food Environment (sections 4-6)<br>Group 3: Seasonal Food Availability Calendar<br>Group 4: Cost of a Healthy Diet<br>Group 5: ProDesirability+<br><br>**Instructions**: Each group will be asked to go over their assigned tool in detail and provide feedback on how it can be improved and any corrections needed.	 Dr. Shauna Downs<br>Dr. Nyda Chhinh<br>Mr. Thou Ponlue<br>Mr. Chen Tepsam Ol<br>Mr. Nayelin Phorn
03:45PM—04:15PM	**Group Presentation (~5 minute each)**<br>Group 1: Personal Food Environment (sections 1-3)<br>Group 2: Personal Food Environment (sections 4-6)<br>Group 3: Seasonal Food Availability Calendar<br>Group 4: Cost of a Healthy Diet<br>Group 5: ProDesirability+<br><br>Questions and answers	Dr. Shauna Downs and Dr. Nyda Chhinh and Mr. Chen Tepsam Ol
04:15PM—04:30PM	Dr. Shauna Downs, Associate Professor<br>Rutgers Global Health Institute	Dr. Serey Sok

Appendix D.

-

Food Environment Toolbox:
Workshop Participant Feedback Survey – Participatory Mapping Focus Group Discussion Guide

The following questions are for the Participatory Mapping Focus Group Guide.

Overall assessment of Participatory Mapping

1. The tool is organized in a way that is clear and understandable for both the facilitator and respondent.

    - Strongly disagree
    - Disagree
    - Neither agree nor disagree
    - Agree
    - Strongly agree

2. Please provide any comments you may have.

3. The language used in the tool is easily comprehended by both the facilitator and respondent.

    - Strongly disagree
    - Disagree
    - Neither agree nor disagree
    - Agree
    - Strongly agree

4. Please provide any comments you may have.

5. The length of the tool is appropriate.

    - Strongly disagree
    - Disagree
    - Neither agree nor disagree
    - Agree
    - Strongly agree

6. Please provide any comments you may have.

7. The tool can be easily used in the field in LMIC settings.

    - Strongly disagree

- Disagree
- Neither agree nor disagree
- Agree
- Strongly agree

8. Please provide any comments you may have.

9. The data obtained from the tool is relevant to LMIC populations.

- Strongly disagree
- Disagree
- Neither agree nor disagree
- Agree
- Strongly agree

10. Please provide any comments you may have.

11. Please provide any comments you may have about the Participatory Mapping Focus Group Discussion Guide overall.

Food Environment Toolbox:
Workshop Participant Feedback Survey – Seasonal Food Availability Calendar Focus Group Discussion Guide

The following questions are for the Seasonal Food Availability Calendar Focus Group Discussion Guide. Keeping the different sections of the tool in mind, please answer the following questions.

Section 4. Sources of listed foods

12. Consumers have the knowledge to determine if the foods they purchase are locally produced or imported.

- Strongly disagree
- Disagree
- Neither agree nor disagree
- Agree
- Strongly agree

13. It is useful to know if consumers believe the foods they purchase are locally produced or imported.

- Strongly disagree
- Disagree
- Neither agree nor disagree
- Agree
- Strongly agree

14. Please provide any additional comments you would like to make about Section 4., sources of listed foods, of the Seasonal Food Availability Calendar Focus Group Discussion Guide.

Section 5. Scoring seasonal availability

15. It is appropriate to begin the scoring of food availability by asking participants which of the free listed items are available throughout the year with little or no variation to streamline the scoring (rather than scoring each food item one by one).

- Strongly disagree
- Disagree
- Neither agree nor disagree
- Agree
- Strongly agree

16. Please provide any additional comments you would like to make about Section 5., scoring seasonal availability, of the Seasonal Food Availability Calendar Focus Group Discussion Guide.

Section 6. Assessing changes in the availability of foods

17. This section includes useful information when assessing changes in the availability of fresh foods available in the community.

- Strongly disagree
- Disagree
- Neither agree nor disagree
- Agree
- Strongly agree

18. Please provide any additional comments you would like to make about Section 6., assessing changes in the availability of foods, of the Seasonal Food Availability Calendar Focus Group Discussion Guide.

Overall assessment of the Seasonal Food Availability Calendar

19. The tool covers all the relevant dimensions of the seasonal availability of fresh foods available in the community.

- Strongly disagree
- Disagree
- Neither agree nor disagree
- Agree
- Strongly agree

20. Please provide any comments you may have.

21. The tool is organized in a way that is clear and understandable for both the facilitator and respondent.

- Strongly disagree
- Disagree
- Neither agree nor disagree
- Agree
- Strongly agree

22. Please provide any comments you may have.

23. The language used in the tool is easily comprehended by both the facilitator and respondent.

- Strongly disagree
- Disagree

- Neither agree nor disagree
- Agree
- Strongly agree

24. Please provide any comments you may have.

25. The length of the tool is appropriate.

- Strongly disagree
- Disagree
- Neither agree nor disagree
- Agree
- Strongly agree

26. Please provide any comments you may have.

27. The tool can be easily used in the field in LMIC settings.

- Strongly disagree
- Disagree
- Neither agree nor disagree
- Agree
- Strongly agree

28. Please provide any comments you may have.

29. The data obtained from the tool is relevant to LMIC populations.

- Strongly disagree
- Disagree
- Neither agree nor disagree
- Agree
- Strongly agree

30. Please provide any comments you may have.

31. Please provide any comments you may have about the Seasonal Food Availability Calendar Focus Group Discussion

Guide overall.

Food Environment Toolbox:
Workshop Participant Feedback Survey – ProDesirability+

Overall assessment of ProDesirability+

32. The tool covers all the relevant information when thinking about capturing the quality of fresh fruits, vegetables, and animal-source foods available for purchase in the community.

- Strongly disagree
- Disagree
- Neither agree nor disagree
- Agree
- Strongly agree

33. Please provide any comments you may have.

34. The tool is organized in a way that is clear and understandable for the enumerator.

- Strongly disagree
- Disagree
- Neither agree nor disagree
- Agree
- Strongly agree

35. Please provide any comments you may have.

36. The criteria for the sensory evaluation of foods (Table 2) is sufficient to enable enumerators to accurately rate the quality of fruits, vegetables, and animal-source foods.

- Strongly disagree
- Disagree
- Neither agree nor disagree
- Agree
- Strongly agree

37. Please provide any comments you may have.

38. The length of the tool is appropriate.

- Strongly disagree
- Disagree
- Neither agree nor disagree
- Agree
- Strongly agree

39. Please provide any comments you may have.

40. The tool can be easily used in the field in LMIC settings.

- Strongly disagree
- Disagree
- Neither agree nor disagree
- Agree
- Strongly agree

41. Please provide any comments you may have.

42. The data obtained from the tool is relevant to LMIC populations.

- Strongly disagree
- Disagree
- Neither agree nor disagree
- Agree
- Strongly agree

43. Please provide any comments you may have.

44. Please provide any comments you may have about the ProDesirability+ tool overall.

Food Environment Toolbox:
Workshop Participant Feedback Survey – Cost of a Healthy Diet Protocol

Please answer the following questions referring to the collection of food price data, as well as the overall assessment as a whole.

Collecting new retail food price data

45. The suggested number of minimum food items to include for each food group is sufficient.

- Strongly disagree
- Disagree
- Neither agree nor disagree
- Agree
- Strongly agree

46. Please provide any comments you may have.

47. The inclusion of discretionary foods (i.e., foods not included in dietary guidelines) is relevant in an LMIC context.

- Strongly disagree
- Disagree
- Neither agree nor disagree
- Agree
- Strongly agree

48. Please provide any additional comments you would like to make about the collection of new price data as part of the Cost of a Healthy Diet protocol.

Overall assessment of Cost of Healthy Diet data collection protocol

49. The tool is organized in a way that is clear and understandable for the enumerator.

- Strongly disagree
- Disagree
- Neither agree nor disagree
- Agree
- Strongly agree

50. Please provide any comments you may have.

51. The language used in the tool is easily comprehended by the enumerator.

- Strongly disagree
- Disagree
- Neither agree nor disagree
- Agree
- Strongly agree

52. Please provide any comments you may have.

53. The length of the tool is appropriate.

- Strongly disagree
- Disagree
- Neither agree nor disagree
- Agree
- Strongly agree

54. Please provide any comments you may have.

55. The tool can be easily used in the field in LMIC settings.

- Strongly disagree
- Disagree
- Neither agree nor disagree
- Agree
- Strongly agree

56. Please provide any comments you may have.

57. The data obtained from the tool is relevant to LMIC populations.

- Strongly disagree
- Disagree
- Neither agree nor disagree
- Agree
- Strongly agree

58. Please provide any comments you may have about the Cost of a Healthy diet protocol overall.

Food Environment Toolbox:
Workshop Participant Feedback Survey – Community Food Environment Mapping Tools

Keeping in mind each of the three tools, please answer the following questions.

Tool 1. Food outlet census: census of all food outlets in community

Section 1. Food outlet properties and food availability

59. This section includes a comprehensive list of the different vendor types people purchase food from.

- Strongly disagree
- Disagree
- Neither agree nor disagree
- Agree
- Strongly agree

60. Please provide any additional comments you would like to make about Section 1., food outlet properties and food availability, of Tool 1. Food Outlet Census as part of the Community Food Environment Mapping exercise.

Section 2. Food and beverage promotion at food outlet

61. This section provides a comprehensive list of the different food and beverage promotions at food outlets in LMICs.

- Strongly disagree
- Disagree
- Neither agree nor disagree
- Agree
- Strongly agree

62. Please provide any comments you may have about Tool 1. Food Outlet Census as part of the Community Food Environment Mapping exercise overall.

Tool 2. Roadside food and beverage promotion: census of roadside promotion of foods and beverages

63. It is important to capture information on roadside promotion of food and beverages.

- Strongly disagree
- Disagree
- Neither agree nor disagree
- Agree
- Strongly agree

64. Please provide any additional comments you would like to make about Tool 2. Roadside Food and Beverage Promotion as part of the Community Food Environment Mapping exercise.

Tool 3. Mobile vendor census: census of mobile vendors that enter the community to sell food

Section 1. Mobile vendor properties and food availability

65. This section includes a comprehensive list of the different mobile vendor types people purchase food from.

- Strongly disagree
- Disagree
- Neither agree nor disagree
- Agree
- Strongly agree

66. Please provide any additional comments you would like to make about Section 1., mobile vendor properties and food availability, of Tool 3. Mobile Vendor Census as part of the Community Food Environment Mapping exercise.

Overall suite of Community Food Environment Mapping tools

67. The list of food groups is appropriate.

- Strongly disagree
- Disagree
- Neither agree nor disagree
- Agree
- Strongly agree

68. Please provide any comments you may have.

69. It is important to elicit information on sub-categories of the provided food groups, such as specifying what types of ultra-processed foods the advertisements are for.

- Strongly disagree
- Disagree
- Neither agree nor disagree
- Agree
- Strongly agree

70. Please provide any comments you may have.

71. It is important to capture information on the form of the foods being sold (e.g., deep fried).

- Strongly disagree
- Disagree
- Neither agree nor disagree
- Agree
- Strongly agree

72. Please provide any comments you may have.

73. It is important to capture information on the state of the foods being sold (e.g., if they are damaged, have insects on them, or are spoiled).

- Strongly disagree
- Disagree
- Neither agree nor disagree
- Agree
- Strongly agree

74. Please provide any comments you may have.

75. The list of promotion types and categories in food outlets is appropriate for LMIC settings.

- Strongly disagree
- Disagree
- Neither agree nor disagree
- Agree
- Strongly agree

76. Please provide any comments you may have.

77. The tools are organized in a way that is clear and understandable for the enumerator.

- Strongly disagree
- Disagree
- Neither agree nor disagree
- Agree
- Strongly agree

78. Please provide any comments you may have.

79. The language used in the tools is easily comprehended by the enumerator.

- Strongly disagree
- Disagree
- Neither agree nor disagree
- Agree
- Strongly agree

80. Please provide any comments you may have.

81. The length of the tools is appropriate.

- Strongly disagree
- Disagree
- Neither agree nor disagree
- Agree
- Strongly agree

82. Please provide any comments you may have.

83. The tools can be easily used in the field in LMIC settings.

- Strongly disagree
- Disagree
- Neither agree nor disagree
- Agree
- Strongly agree

84. Please provide any comments you may have.

85. The data obtained from the tools is relevant to LMIC populations.

- Strongly disagree
- Disagree
- Neither agree nor disagree
- Agree
- Strongly agree

86. Please provide any comments you may have about the set of Community Food Environment Mapping tools overall.

Food Environment Toolbox:

Workshop Participant Feedback Survey – Market Mapping Modules

Keeping in mind each of the modules, please answer the following questions.

Module 1. Market properties and infrastructure: overview of the market

87. The section includes a comprehensive list of different kinds of markets that people have access to.

- Strongly disagree
- Disagree
- Neither agree nor disagree
- Agree
- Strongly agree

88. Please provide any comments you may have.

89. The tool captures sufficient information about the market properties.

- Strongly disagree
- Disagree
- Neither agree nor disagree
- Agree
- Strongly agree

90. Please provide any additional comments you would like to make about Module 1., market properties and infrastructure, of the Market Mapping exercise.

Module 2. Market vendor roster: census of all vendors in the market

91. It is important to collect information on every vendor in the market.

- Strongly disagree
- Disagree
- Neither agree nor disagree
- Agree
- Strongly agree

92. Please provide any comments you may have.

93. This section includes a comprehensive list of the different vendor types people purchase food from.

- Strongly disagree
- Disagree
- Neither agree nor disagree
- Agree
- Strongly agree

94. Please provide any comments you may have.

95. Please provide any additional comments you would like to make about Module 2., market vendor roster, of the Market Mapping exercise.

Market 3. Market promotion: census of all promotions of foods and beverages at the market

96. The list of promotion types and categories in markets is appropriate for LMIC settings.

- Strongly disagree
- Disagree
- Neither agree nor disagree
- Agree
- Strongly agree

97. Please provide any additional comments you would like to make about Module 3., market promotion, of the Market Mapping exercise.

Overall assessment of the Market Mapping Tool

98. The tool covers all the relevant dimensions of the market.

- Strongly disagree
- Disagree
- Neither agree nor disagree
- Agree
- Strongly agree

99. Please provide any comments you may have.

100. The modules are organized in a way that is clear and understandable for the enumerator.

- Strongly disagree
- Disagree
- Neither agree nor disagree
- Agree
- Strongly agree

101. Please provide any comments you may have.

102. The language used in the modules is easily comprehended by the enumerator.

- Strongly disagree
- Disagree
- Neither agree nor disagree
- Agree
- Strongly agree

103. Please provide any comments you may have.

104. The length of the modules is appropriate.

- Strongly disagree
- Disagree
- Neither agree nor disagree
- Agree
- Strongly agree

105. Please provide any comments you may have.

106. The modules can be easily used in LMIC settings.

- Strongly disagree

- Disagree
- Neither agree nor disagree
- Agree
- Strongly agree

107. Please provide any comments you may have.

108. The data obtained from the modules is relevant to LMIC populations.

- Strongly disagree
- Disagree
- Neither agree nor disagree
- Agree
- Strongly agree

109. Please provide any comments you may have about the Market Mapping modules overall.

Food Environment Toolbox:
Workshop Participant Feedback Survey – In-Depth Vendor Assessment

The following questions are for the In-depth Vendor Assessment. Keeping in mind each section of the tool, please answer the following questions.

Section 1. Vendor properties

110. The section includes a comprehensive list of different vendor types that people have access to.

- Strongly disagree
- Disagree
- Neither agree nor disagree
- Agree
- Strongly agree

111. Please provide any comments you may have.

112. Please provide any additional comments you would like to make about Section 1., vendor properties, of the In-Depth Vendor Assessment.

Section 3. Labeling

113. This section elicits sufficient information when asking about labeling on different food groups.

- Strongly disagree
- Disagree
- Neither agree nor disagree
- Agree
- Strongly agree

114. Please provide any comments you may have.

115. The list of information or labeling that can be seen on food packaging is appropriate.

- Strongly disagree
- Disagree

- Neither agree nor disagree
- Agree
- Strongly agree

116. Please provide any additional comments you would like to make about Section 3., labeling, of the In-Depth Vendor Assessment.

Section 4. Sustainability

117. This section elicits sufficient information when asking about aspects of sustainability related to the vendor.

- Strongly disagree
- Disagree
- Neither agree nor disagree
- Agree
- Strongly agree

118. Please provide any additional comments you would like to make about Section 4., sustainability, of the In-Depth Vendor Assessment.

Section 6. Hygiene

119. This section elicits sufficient information when asking about vendor hygiene.

- Strongly disagree
- Disagree
- Neither agree nor disagree
- Agree
- Strongly agree

120. Please provide any additional comments you would like to make about Section 6., hygiene, of the In-Depth Vendor Assessment.

Overall assessment of In-Depth Vendor Assessment

121. The tool covers all the relevant dimensions when observationally evaluating a vendor.

- Strongly disagree
- Disagree
- Neither agree nor disagree
- Agree
- Strongly agree

122. Please provide any comments you may have.

123. The tool is organized in a way that is clear and understandable for the enumerator.

- Strongly disagree
- Disagree
- Neither agree nor disagree
- Agree
- Strongly agree

124. Please provide any comments you may have.

125. The language used in the tool is easily comprehended by the enumerator.

- Strongly disagree
- Disagree
- Neither agree nor disagree
- Agree
- Strongly agree

126. Please provide any comments you may have.

127. The length of the tool is appropriate.

- Strongly disagree
- Disagree
- Neither agree nor disagree
- Agree
- Strongly agree

128. Please provide any comments you may have.

129. The tool can be easily used in the field in LMIC settings.

- Strongly disagree
- Disagree
- Neither agree nor disagree
- Agree
- Strongly agree

130. Please provide any comments you may have.

131. The data obtained from the tool is relevant to LMIC populations.

- Strongly disagree
- Disagree
- Neither agree nor disagree
- Agree
- Strongly agree

132. Please provide any comments you may have about the In-Depth Vendor Assessment overall.

Food Environment Toolbox:
Workshop Participant Feedback Survey – Personal Food Environment Survey Instrument

Keeping in mind each section of the tool, please answer the following questions.

Section 1. Accessibility

133. The tool includes a comprehensive list of different food environments that people have access to.

- Strongly disagree
- Disagree
- Neither agree nor disagree
- Agree
- Strongly agree

134. Please provide any comments you may have.

135. Please provide any additional comments you would like to make about Section 1., accessibility, of the Personal Food Environment Survey Instrument.

Section 2. Affordability

136. This section elicits sufficient information when asking about the affordability of different food groups.

- Strongly disagree
- Disagree
- Neither agree nor disagree
- Agree
- Strongly agree

137. Please provide any additional comments you would like to make about Section 2., affordability, of the Personal Food Environment Survey Instrument.

Section 3. Convenience

138. This section elicits sufficient information when asking about the convenience of different food groups.

- Strongly disagree

- Disagree
- Neither agree nor disagree
- Agree
- Strongly agree

139. Please provide any additional comments you would like to make about Section 3., convenience, of the Personal Food Environment Survey Instrument.

Section 4. Quality and safety

140. This section includes elicits sufficient information when asking about the quality and safety of different food groups.

- Strongly disagree
- Disagree
- Neither agree nor disagree
- Agree
- Strongly agree

141. Please provide any additional comments you would like to make about Section 4., quality and safety, of the Personal Food Environment Survey Instrument.

Section 5. Promotion and labeling

142. This section includes elicits sufficient information when asking about the promotion and labeling of different food groups.

- Strongly disagree
- Disagree
- Neither agree nor disagree
- Agree
- Strongly agree

143. Please provide any additional comments you would like to make about Section 5., promotion and labeling, of the Personal Food Environment Survey Instrument.

Section 6. Sustainability

144. This section includes elicits sufficient information when asking about the sustainability of foods over time in their community.

- Strongly disagree
- Disagree
- Neither agree nor disagree
- Agree
- Strongly agree

145. Please provide any additional comments you would like to make about Section 6., sustainability, of the Personal Food Environment Survey Instrument.

Overall assessment of the Personal Food Environment Survey Instrument

146. The tool covers all the relevant dimensions of the personal food environment.

- Strongly disagree
- Disagree
- Neither agree nor disagree
- Agree
- Strongly agree

147. Please provide any comments you may have.

148. The tool is organized in a way that is clear and understandable for both the enumerator and respondent.

- Strongly disagree
- Disagree
- Neither agree nor disagree
- Agree
- Strongly agree

149. Please provide any comments you may have.

150. The language used in the tool is easily comprehended by both the enumerator and respondent.

- Strongly disagree
- Disagree
- Neither agree nor disagree

- Agree
- Strongly agree

151. Please provide any comments you may have.

152. The length of the tool is appropriate.

- Strongly disagree
- Disagree
- Neither agree nor disagree
- Agree
- Strongly agree

153. Please provide any comments you may have.

154. The tool can be easily used in the field in LMIC settings.

- Strongly disagree
- Disagree
- Neither agree nor disagree
- Agree
- Strongly agree

155. Please provide any comments you may have.

156. The data obtained from the tool is relevant to LMIC populations.

- Strongly disagree
- Disagree
- Neither agree nor disagree
- Agree
- Strongly agree

157. Please provide any comments you may have about the personal food environment survey instrument overall.
